# Novel *VARS1* variants define new clinical and molecular subtypes of a rare neurodevelopmental syndrome

**DOI:** 10.1016/j.bbadis.2026.168184

**Published:** 2026-02-10

**Authors:** Busra Aynekin, Tracy Lau, Rauan Kaiyrzhanov, Ioannis Papazoglou, Ayten Gulec, Ummu Gulsum Ozgul Gumus, Svetlana Gorokhova, Busa Tiffany, Leonardo Simão Medeiros, Ida Vanessa Doederlein Schwartz, Mehmet Burak Mutlu, Sofia Ourani, Anke Bergman, Kelly Schoch, Huseyin Per, Nurdeniz Nalbant Bingol, Sehime G. Temel, Serdar Durdağı, Semra Hız, Geneviève Bernard, Felipe Villa Tobon, Henry Houlden, Stephanie Efthymiou

**Affiliations:** a Department of Neuromuscular Disorders, UCL Queen Square Institute of Neurology, London, WC1N 3BG, UK; b Department of Molecular Biology and Genetics, Biruni University, 34015, Istanbul, Türkiye; c Laboratory for Innovative Drugs (Lab4IND), Computational Drug Design Center (HİTMER), Bahçeşehir University, 34734, İstanbul, Türkiye; d Department of Pediatrics, Erciyes University, Faculty of Medicine, Kayseri, Turkey; e Department of Medical Genetics, Timone Children’s Hospital, APHM, Marseille, France; f Medical Genetics Service-Hospital de Clinicas de Porto Alegre-Brazil, Porto Alegre, RS, Brazil; g Department of Medical Genetics, Detagen Genetic Diseases Evaluation Center, Kayseri, Türkiye; h Clinical Genetics Department, Archbishop Makarios III Hospital, Nicosia, Cyprus; i Hannover Medical School, Institute of Human Genetics, Hannover, Germany; j Division of Medical Genetics, Pediatrics, Duke University School of Medicine, Durham, NC, USA; k Department of Translational Medicine, Institute of Health Sciences, Bursa Uludag University, Bursa, Türkiye; l Department of Medical Genetics, Faculty of Medicine, Bursa Uludag University, Bursa, Türkiye; m Department of Histology and Embryology, Faculty of Medicine, Bursa Uludag University, Bursa, Türkiye; n Molecular Therapy Lab, Department of Pharmaceutical Chemistry, School of Pharmacy, Bahcesehir University, Istanbul, Türkiye; o Systems Biology Lab, Biruni University Scientific Research Center, Biruni University, Istanbul, Türkiye; p Department of Pediatric Neurology, Faculty of Medicine, Dokuz Eylül University, Izmir, 35340, Turkey; q Department of Neurology and Neurosurgery, McGill University, Montreal, Canada; r Departments of Pediatrics and Human Genetics, McGill University, Montreal, Canada; s Child Health and Human Development Program, McGill University Health Centre Research Institute, Montreal, Canada; t Department Specialized Medicine, Division of Medical Genetics, McGill University Health Centre, Montreal, Canada; u Department of Human Genetics, McGill University, Montreal, Canada

**Keywords:** Monogenic diseases, Mendelian disorders, Aminoacyl tRNA synthetase, Molecular dynamics, Neurodevelopment

## Abstract

**Purpose:**

We aimed to broaden the understanding of autosomal recessive neurodevelopmental disorders caused by *VARS1* by describing new clinical and molecular findings and assessing the predicted structural impact of identified variants.

**Methods:**

We clinically evaluated 13 affected individuals from 10 unrelated families presenting with a neuro-developmental disorder. We used exome sequencing and cosegregation analyses to identify disease-causing variants, followed by three-dimensional *in silico* analyses and molecular dynamics simulations to assess the likely functional consequences of both previously reported and novel variants.

**Results:**

In all affected individuals who presented with a neurodevelopmental syndrome with progressive microcephaly, seizures, and intellectual disability, we identified biallelic disease-causing variants in *VARS1*. Two variants were predicted to induce premature protein truncation leading to loss of VARS1 function. The remaining 13 detected missense variants were located in the catalytic and aminoacylation domains, and *in silico* analysis of the affected residues showed that such substitutions can disrupt local protein dynamics, RNA-interaction surfaces, or catalytic geometry, thereby affecting ligand recognition, substrate specificity, and tRNA interaction.

**Conclusion:**

Together with prior reports, our results provide strong additional evidence supporting *VARS1* as a recurrent cause of autosomal recessive neurodevelopmental disorders and expand the known clinical and allelic spectrum. While *in silico* analyses provide mechanistic plausibility for novel variants, functional studies will be important to confirm variant-specific effects and disease mechanisms.

## 1 Introduction

Aminoacyl-tRNA synthetases (ARSs) are crucial enzymes in protein synthesis, ensuring accurate pairing of amino acids to their specific tRNA molecules [1,2]. At present, there are 37 ARS genes known in humans, with 18 functioning in the cytoplasm, 17 in the mitochondria, and 2 that serve both compartments [3]. Pathogenic mutations in 31 *ARS* genes have been associated with neurodevelopmental disorders [4], cancer [5], and autoimmune diseases [6]. *Valyl-tRNA synthetase 1* (*VARS1*) (MIM: 192150) encodes an enzyme that is fundamental in the attachment of valine to valine-tRNA [7]. Pathogenic mutations in *VARS1* are associated with ‘Neurodevelopmental disorder with microcephaly, seizures, and cortical atrophy’ (NDMSCA) (MIM:617802) [8]. Karaca et al. first reported two pathogenic variants, NM_006295.2: c.2653C>T p.(Leu885Phe) and c.3173G>A p.(Arg1058Gln), in two consanguineous families with epileptic encephalopathy [9]. Additional variants in patients with similar clinical findings are associated with biallelic variants in *VARS1* [10–14].

*In vitro* studies using patient-derived cell lines, including enzymatic and yeast complementation assays, indicate that recessive *VARS* mutations likely result in a loss-of-protein function. This is consistent with them being loss-of-function (LoF) alleles. Furthermore, a *VARS1* knockout zebrafish model mirrors key human disease characteristics, exhibiting microcephaly and epileptiform activity [12]. In this study, we identified a total of 16 different variants in 10 unrelated families, this includes 10 variants not previously documented in existing literature – eight missense (p.(Leu16Arg), p.(Pro51Ser), p.(Met296Val), p. (Arg404Gln), p.(His566Tyr), p.(Arg665Trp), p.(Ser866Cys), and p. (Asp920Asn)), one synonymous (p.(Leu631)) and one premature stop codon (p.(Trp790*). This work contributes to a broader understanding of the *VARS1* mutational spectrum and elucidates the genotype-phenotype correlation in NDMSCA.

## 2 Material and methods

### 2.1 Participants and clinical investigations

Families were recruited through international collaborations and the use of GeneMatcher. The identification of the seven families described in this report involved exome sequencing (ES) and the collaborative sharing of data between genetic centers worldwide, with GeneMatcher being instrumental in patient recruitment for this study. The study was approved by the Research Ethics Committee Institute of Neurology University College London (IoN UCL) (07/Q0512/26) and the local Ethics Committees of each participating center.

### 2.2 Exome sequencing

Genomic DNA was extracted from peripheral blood samples, according to standard procedures of phenol-chloroform extraction. Exome sequencing on DNA of subjects was performed as described elsewhere in Macrogen, Korea (UCL) or at collaborating centers. Briefly, target enrichment was performed with 2 μg genomic DNA using the Sure-SelectXT Human All Exon Kit version 6 (Agilent) to generate barcoded exome sequencing libraries. Libraries were sequenced on the HiSeqX platform (Illumina) with 50× coverage. Quality assessment of the sequence reads was performed by generating QC statistics with FastQC. The bioinformatics filtering strategy included screening for only exonic and donor/acceptor splicing variants [15].

### 2.3 Evolutionary-guided sequence analysis

#### 2.3.1 Conservation analysis and multiple sequence alignment (MSA)

The full-length protein sequence (UniProt ID: P26640) was analyzed for evolutionary conserved residues using Multiple Sequence Alignment (MSA) with the ConSurf web server [16–20]. Homologous sequences were taken from UniProt90, filtered for 35–95% identity, and aligned with MAFFT-L-INS-i to compute Bayesian conservation scores [21–23].

#### 2.3.2 Domain annotation and structural inference based on homology

To determine the structural elements and functional domains of human valyl-tRNA synthetase, we first used InterPro to acquire sequence-based annotations [24]. We used the high-resolution bacterial valyl-tRNA synthetase structures from the PDB because there was no experimental human protein structure available [25–27]. By using BLASTp alignment to map the knowledge from these homologous structures onto the human sequence, functional features could then be interpreted at the residue level [28].

### 2.4 Structure acquisition, in silico mutagenesis and molecular dynamics simulations

In the absence of an experimental structure, the human valyl-tRNA synthetase was modeled using AlphaFold2 (apo state) and AlphaFold3 (post-aminoacylation complex with tRNA-Val and AMP, see also details in section 1.3 of Supplementary Material). The human valyl-tRNA synthetase apo-structure predicted by AlphaFold2 (AF2) was simulated for 200 ns using all-atom molecular dynamics (MD) simulation in physiological conditions [29–37]. In order to assess each mutant’s impact on protein stability and dynamics, novel and disease-associated point mutations were introduced *in silico*. All simulations were conducted using GROMACS 2023.3 [38,39]. The details of the MD simulation settings are provided in section 1.3 of Supplementary Materials.

The approach of performing 200 ns MD simulations, while relatively short for such large systems (~500,000 atoms each), was chosen based on our computational constraints and the research questions being addressed: here, we aimed for an initial characterization of mutation effects on ValRS stability and dynamics. Recent studies investigating mutation effects on protein dynamics have successfully employed similarly short simulations (100 ns) per variant to capture residue-level flexibility changes []. We acknowledge, however, that longer timescales are required to capture complete mutation-induced conformational transitions.

Due to the same computational constraints, we had limited ability to perform independent replica runs for all systems, however, we validated our single-trajectory results by performing triplicate simulations for four representative systems (wild-type, R404Q, H566Y, D920N). While multiple independent replicas are generally recommended to ensure reproducibility in MD studies [], we made a practical trade-off, acknowledging that for our specific analyses (local flexibility, root mean square fluctuation, betweenness centrality) single well-converged trajectories provide meaningful initial insights. Eventually, the consistency of our results across the replicated systems (Tables S13-S14) along with the RMSD convergence observed in our systems (Figs. S3–7) supported the reliability of our single-trajectory analyses to provide an initial characterization of these novel identified mutations.

### 2.5 Trajectory analysis

#### 2.5.1 Root Mean Square Deviation (RMSD) and Root Mean Square Fluctuation (RMSF)

Structural stability was assessed using RMSD analysis of protein backbone atoms, calculated per individual domain using Visual Molecular Dynamics (VMD). VMD was also used to perform per-residue RMSF analysis to measure how much each residue fluctuates during the simulation. To identify mutation effects, we calculated ΔRMSF by subtracting mutant RMSF values from the wild-type ones. Thus, positive ΔRMSF values indicate that the residue is less flexible in the mutant, while negative ΔRMSF values indicate that the residue is more flexible in the mutant.

#### 2.5.2 Principal component analysis (PCA) and clustering

Large-scale collective motions within the protein’s enzymatic core were characterized by applying PCA to the MD trajectories [40]. Using scikit-learn’s PCA class on Cα atom coordinates, we were able to identify dominant conformational motions and sample conformational clusters of the AF2-predicted apo structure.

#### 2.5.3 Network-based analysis of residue communication

Betweenness centrality (BC) was computed from residue interaction networks produced by MD-TASK in order to evaluate intra-protein communication [41]. BC quantifies how frequently a residue lies on the shortest paths connecting other residue pairs, thereby identifying communication hubs critical for signal transfer through the protein structure. By subtracting the mutant BC values from the wild-type ones, we identified mutation-induced disruptions in functional communication pathways. Positive ΔBC values indicate that the residue has reduced hub importance in the mutant, while negative ΔBC values indicate that the residue has increased hub importance in the mutant. More information regarding BC and how it is measured can be found in section 1.4.3 of the Supplementary Material.

#### 2.5.4 Dynamic cross-correlation analysis of residue motions

Residue-residue coordination was measured using Dynamic Cross-Correlation Maps (DCCMs) [42,43]. The identification of mutation-induced alterations in dynamic coupling within functional domains was made possible by DCCMs, which were calculated using MD-TASK [41]. More information can be found in section 1.4.4 of the Supplementary Material.

#### 2.5.5 Dynamic binding analysis throughout trajectory

We used a custom dynamic docking pipeline that directly integrated with MD trajectories in order to assess the effect of mutations on ligand binding [44,46]. Time-resolved binding affinity and ligand pose stability can be measured thanks to this workflow, which automates frame extraction, receptor preparation, and ligand docking. By monitoring dynamic changes in the ATP-valyl substrate interaction, this analysis evaluated how mutations might impair the protein’s capacity to charge tRNA. Methodological details can be found in section 1.4.5 of the Supplementary Material.

## 3 Results

In total, 7 males and 4 females from 10 different families were identified (Clinical details in Table S1). The mean age at last examination was 3 years (min: 1.5 years, max: 8 years) (median: 3 years). At present, all patients are alive except two, where one of the patients passed away due to complications caused by an upper respiratory infection. In 2/13 of patients, premature delivery was detected. Regardless of prematurity, intrauterine growth restriction (IUGR) was observed in 3/13 of patients. Seven patients were presented with progressive microcephaly, one patient (subject 9) was described to have a smaller head size compared to other growth parameters, indicating microcephaly, though it was not considered progressive. Most patients assessed with age-appropriate testing had profound delayed psychomotor development or severe intellectual disability. None of the patients were able to speak; some were able to make a sound. Although subject 5 had glaucoma, 3/13 of the other patients had no eye contact and optic atrophy. Except the subjects 8 and 9, all the patients developed early-onset seizures (average age of onset 3–6 months) - often myoclonic, refractory seizures were reported. Progressive cortical and cerebellar atrophy on brain MRI was reported in 5/13 of patients. Decreased white matter and a thin corpus callosum were present in 3/13 of the patients. Two patients had feeding problems requiring feeding tubes (one had percutan gastrostomy tube). The two siblings (family 7) presenting with a relatively milder phenotype presented with atypical autism and sleep problems needing medical therapy. Despite the presence of marked hypotonicity, none of the patients required invasive mechanical ventilation. 2/13 of the patients had multiple cardiac involvements, including pericardial effusion, cardiac thrombus, mass lesion, patent ductus arteriosus, atrial septal defect, ventricular septal defect.

### 3.1 ES reveals novel and known biallelic variants in VARS1

**Fig. 1:**
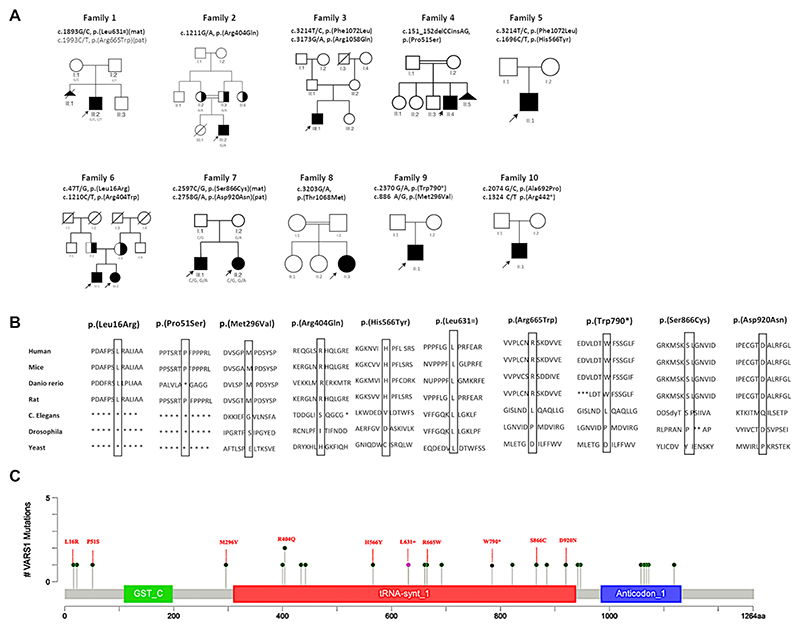
A. Pedigrees of ten unrelated families carrying biallelic *VARS1* variants. Filled symbols indicate affected individuals; arrows mark probands. Half-filled symbols represent heterozygous carriers or individuals with unknown phenotype, while slashed symbols indicate deceased individuals. B. Schematic representation of the domain architecture of the human VARS1 protein and distribution of identified variants. Red-labeled variants are novel ten variants identified in this study, while others were previously reported and also found in our cohort. Variants are colour-coded based on their type: green dots represent missense mutations, the black dot represents a truncating mutation, and the pink dot denotes another type of mutation. The schematic illustrates the domain architecture, including the Glutathione S-transferase C-terminal (GST_C) domain (green), tRNA synthetase catalytic (tRNA-synt_1) domain (red), and the Anticodon-binding domain (blue). Vertical lines represent the position and corresponding name of each variant mapped onto the VARS1 protein [45]. C. The conservation analysis of ten novel *VARS1* variants across species. The alignment shows the conservation of amino acid residues corresponding to the identified *VARS1* variants across multiple species. (For interpretation of the references to colour in this figure legend, the reader is referred to the web version of this article.)

ES of affected individuals identified a total of sixteen coding variants in *VARS1* (Fig. 1). These include synonymous, missense and premature termination codon. Variants detected in this study are as follows (in order of family number): c.1993C > T, p.(Arg665Trp) (**R665W**); c.1893G > C, p.(Leu631=); **(L631=)**, c.1211G > A, p.(Arg404Gln) (**R404Q**); c.3214 T > C, p.(Phe1072Leu) (**F1072L**); c.3173G > A, p. (Arg1058Gln) (**R1058Q**); c.151_152delCCinsAG, p.(Pro51Ser) (**P51S**); c.1696C > T, p.(His566Tyr) (**H566Y**); c.47 T > G, p.(Leu16Arg) (**L16R**); c.1210C > T, p.(Arg404Trp) (**R404W**); c.2597C > G, p.(Ser866Cys) (**S866C**); c.2758G > A, (p.Asp920Asn) (**D920N**); c.3203G > A, p. (Thr1068Met) (T1068M); c.2370G > A, p.(Trp790*) (W790*), c.886 A > G, p.(Met296Val) (**M296V**); c.3214 T > C, p.(Ala692Pro) (**A692P**), and c.1324C > T, p.(Arg442*) (R442*). Each variant has been assigned an internal abbreviated label (*e.g*., R665W, R404Q), which will be used throughout this manuscript. All variants discovered in this study had no to very low allele frequency in numerous population databases (~2 mln alleles).

Among the identified genetic changes, ten variants (L16R, P51S, M296V, R404Q, H566Y, L631=, R665W, W790*, S866C, and D920N) were considered novel. The majority of the novel variants were located in the aminoacylation (tRNA-synt_1) domain (Fig. 1B) and were highly conserved across different species (Fig. 1C). For the missense variants (*n* = 8), they became subjects of computational modeling and all atom MD simulations in this work. Two of the novel variants, the synonymous L631 = and the premature stop codon W790*, were excluded from *in silico* structural analyses as the synonymous variant is functionally equivalent to the wild-type (WT), and the premature stop codon variant (p.Trp790*) could not be structurally modeled due to loss of C-terminal structural domains.

The remaining variants (R404W, R442*, A692P, R1058Q, T1068M, F1072L) represent previously reported pathogenic missense mutations associated with early-onset neurodevelopmental phenotypes, including microcephaly, epileptic encephalopathy, and intellectual disability [11,14] (Table S1). Among the known mutations, they reduce enzymatic activity, resulting in either a complete or partial loss of function (A692P, R1058Q, T1068M, F1072L). Other mutations significantly alter the enzyme structure or prevent protein expression entirely (R404W, R442*) [11]. As such, they form an important benchmark for comparing the effects of newly identified mutations to impair distinct aspects of ValRS structure or function.

### 3.2 Evolutionary conservation of mutated residues

The residues carrying the novel substitution mutations identified in section 3.2 exhibit varying degrees of evolutionary conservation, as demonstrated by MSA analysis. The alignment included a representative panel of 500 homologous sequences derived from a comprehensive set of 117,089 homologues extracted from the UNIREF90 database (see methods). Residues Arg404 (conservation score: –1.021), His566 (–1.057), Arg665 (–1.117), Ser866 (–1.150), and Asp920 (–1.214) display high evolutionary conservation. Specifically, Asp920 is conserved as aspartic acid in 99% of sequences (<1% His), Ser866 as serine in 96% of sequences, and Arg404 as arginine in 92% of sequences (Table 1). Such minimal variability underscores strong purifying selection pressures acting on these residues. His566 shows a somewhat more permissive substitution profile (52% Leu, 42% His), suggesting a constrained local environment favoring hydrophobic packing. Conservation scores till now imply that mutations at these highly conserved positions are likely to substantially impact the protein’s structure and function. On the contrary, Met296 exhibits lower evolutionary constraint, reflected by a lower conservation score (–0.153) and a broad spectrum of tolerated amino acid residues (39% Leu, 32% Met, 10% Phe, 3% Tyr), suggesting less functional impact unless the substitution drastically alters local packing. Furthermore, residue Pro51 (0.639; 60% Pro, 20% His or Asn) also exhibit greater variability, indicative of relaxed evolutionary constraints, consistent with its localization in the eukaryote-specific N-terminal domain. In contrast, Leu16 (conservation score: -0.467; 94% Leu, 5% Ala) is less variable (Table 1). To ensure result robustness, the MSA was verified for reliability by examining alignment of the known highly conserved motif KMSKS (residues 862–866), which indeed existed in perfect alignment and presented high conservation (MSA Data: >499/500, Table S4), thus supporting the validity of the presented evolutionary insights.

### 3.3 Acquisition and domain annotation of human ValRS structure

**Fig. 2:**
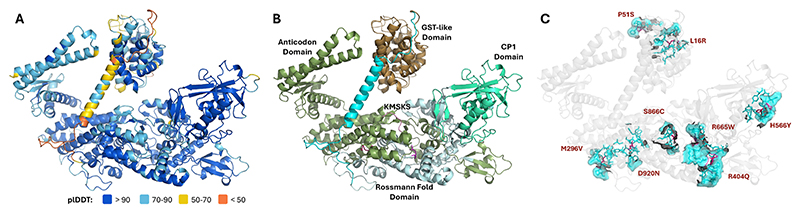
AlphaFold2-predicted apo-state structure of the human ValRS (AF-P26640-F1-v4). A. Predicted structure colored by AlphaFold2 confidence score (pLDDT: 88.28), B. Like other class Ia aminoacyl-tRNA synthetases, ValRS comprises of four major domains: the GST-like domain (eukaryotic-specific), CP1 editing domain, Rossmann fold domain and anticodon-binding domain. The highly conserved KMSKS catalytic motif, which controls amino acid entry prior to tRNA charging, is shown in pink, C. This study characterizes eight novel ValRS mutations: two in the GST-like domain (L16R, P51S), five in the Rossmann fold and anticodon-binding domains (M296V, R404Q, R665W, S866C, D920N) and one in the CP1 editing domain (H566Y). (For interpretation of the references to colour in this figure legend, the reader is referred to the web version of this article.)

**Fig. 3:**
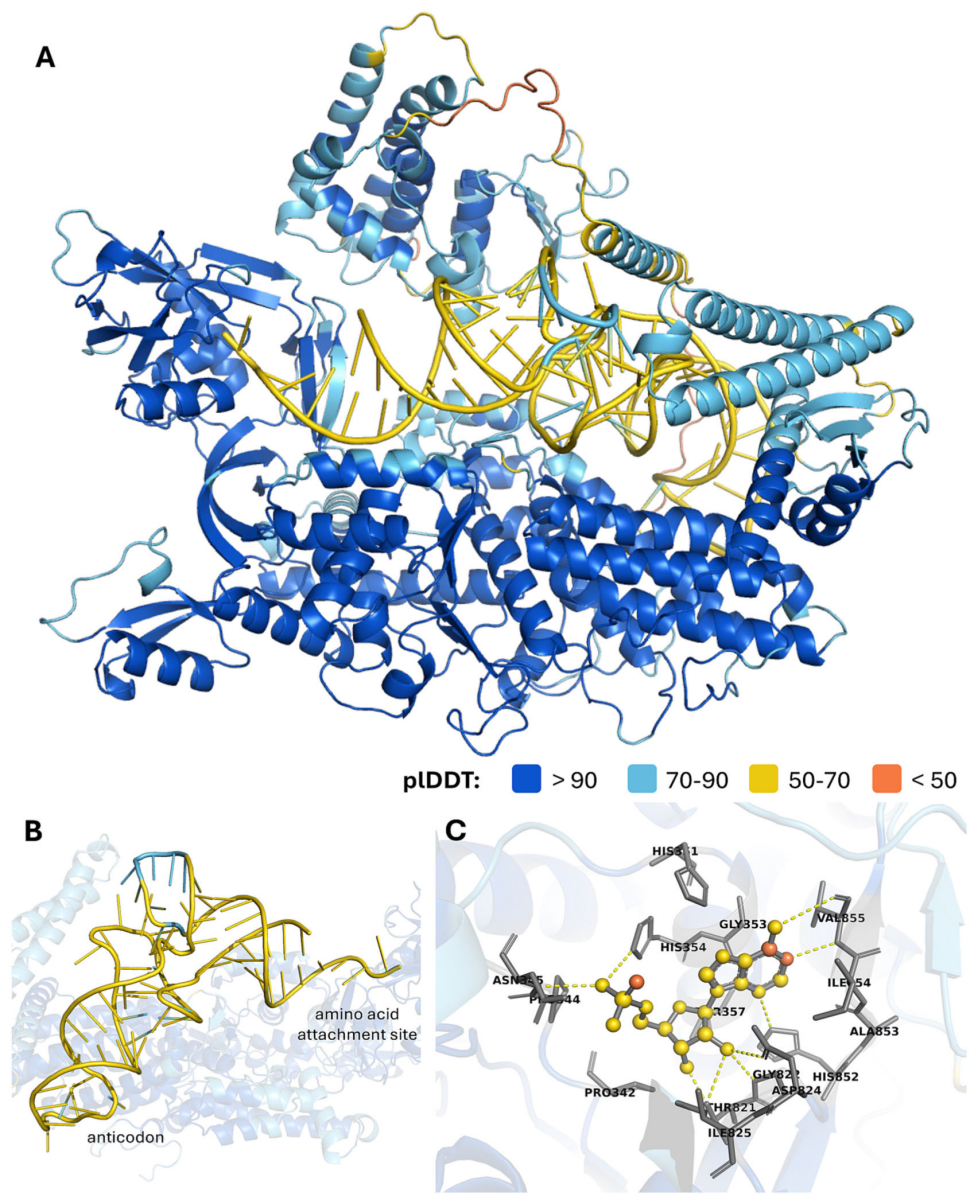
AlphaFold3-predicted holo-state structure of human ValRS in complex with tRNA and AMP, colored by confidence score (pLDDT: Cα for protein, C4’ for RNA). A. The predicted protein structure shows high overall confidence (89.33 pLDDT), however, residues at the tRNA interface exhibit reduced confidence, B. The predicted tRNA structure shows 65.71 pLDDT confidence, C. Adenosine monophosphate (AMP) positioning shows 57.82 pLDDT confidence, however with several surrounding residues consistent with expected binding site contacts (see Table S5).

Due to the lack of an experimentally resolved structure for the human ValRS enzyme, we aimed to curate a reliable three-dimensional model that could serve as a foundation for downstream functional analysis. Thus, initially, the predicted apo-structure from the Alpha-Fold2 database (AF-P26640-F1-v4) was curated presenting high predicted structural confidence (pLDDT): core catalytic Rossmann fold (94.13 ± 4.38), CP1 editing domain (94.95 ± 4.78), anticodon-binding domain (90.94 ± 6.78) and GST-like domain (80.64 ± 17.08) (Fig. 2A-B). To interpret the model, we used domain annotations deriving from InterPro database: core catalytic Rossmann fold (UniProt residues 282–494, 655–734) and CP1 editing domain (495–654), anticodon-binding domain (735–1264) and a eukaryotic-specific GST-like domain at the N-terminus (1–218) (Fig. 2C). Sequentially, we employed AlphaFold3 to predict the full-length human ValRS structure bound to the adenosine monophosphate (AMP) substrate and tRNA (tRNA-Val-TAC-1-1 + CCA). While high-confidence structural predictions were obtained for the protein itself (pLDDT for Rossmann fold: 94.47 ± 3.38, CP1: 93.75 ± 2.99, anticodon-binding domain: 91.08 ± 7.13 and GST-like domain: 82.96 ± 11.80), the confidence score for the AMP (pLDDT: 57.82 ± 5.80) and the tRNA (pLDDT: 65.71 ± 4.03) structure and positioning were notably lower (Fig. 3).

To validate the structural accuracy of the protein component, we aligned both predicted models to the crystal structure of the bacterial ortholog from *Thermus thermophilus* (PDB code: 1GAX [25]. This analysis confirmed strong structural conservation across species (Fig. S1A), with near-identical side-chain geometries at key residues involved in aminoacylation, editing and anticodon recognition (Figs. S1B-E). However, despite favorable alignment of the protein, we decided to proceed with only the apo structure for MD simulations. This decision was based on considerations: (i) the AlphaFold3 holo model showed substantially lower confidence for the tRNA and substrate components; (ii) the positioning of the tRNA in the predicted holo complex lacks experimental validation and could introduce artifactual biases into the simulations; and (iii) by simulating the apo-state we opted to directly assess how each point mutation affects intrinsic protein stability and dynamics, independent of binding-induced conformational effects.

### 3.4 Refinement of AlphaFold predicted structure using MD SIMULATIONS

While AlphaFold2 provides overall high-confidence structural predictions, its models represent static, energy-minimized conformations that lack information about natural fluctuations and solvent-induced dynamics. To bring our structure closer to biologically realistic behavior and identify stable conformational states, we refined the AF2-predicted apo-state structure of human ValRS using all-atom MD simulations. Initially, we performed three independent 200 ns MD simulations in explicit solvent (TIP3P water box and 0.15 M NaCl), allowing the protein to explore its conformational energy landscape under near-physiological conditions. For the post-simulation analysis, two parts of the protein were used examined separately: the N-terminal GST-like domain (residues 1–218) and the catalytic region (residues 282–1264), in order to exclude signal noise from the highly flexible interdomain linker.

**Fig. 4:**
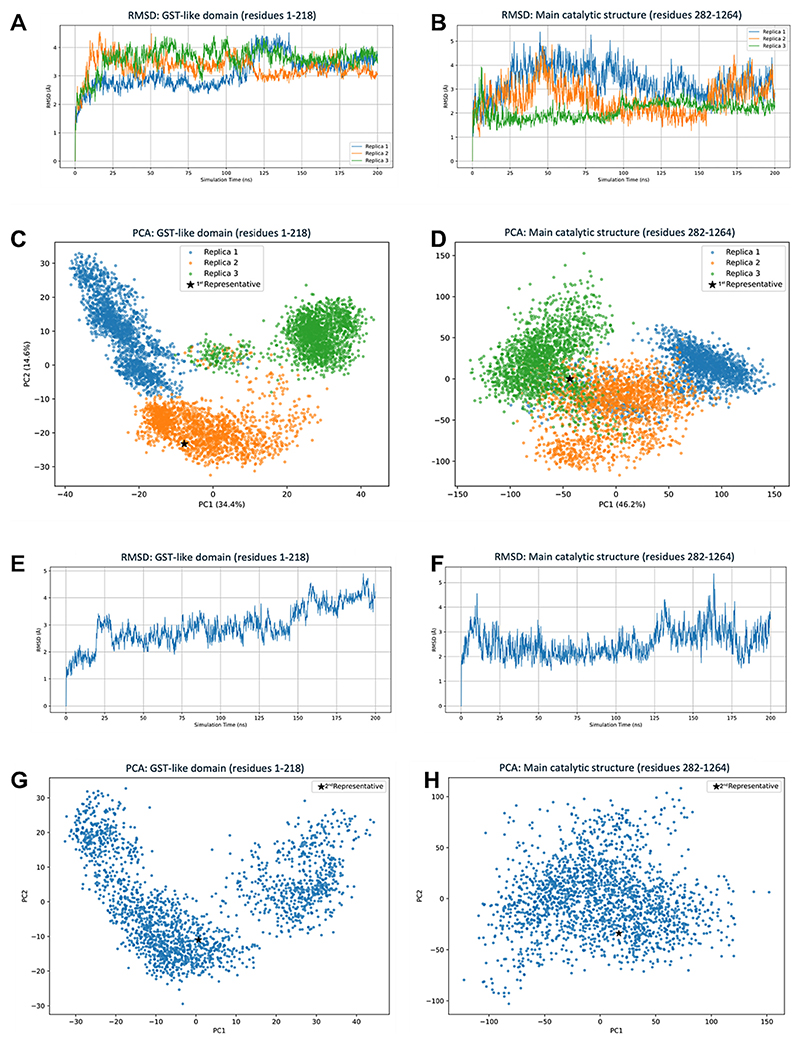
Iterative MD simulation workflow for refining ValRS structure from the AlphaFold2 prediction. Three independent 200 ns replica MD simulations were initiated from the AlphaFold2-predicted structure (A-D): A. RMSD progression for the GST-like domain, B. RMSD progression for the catalytic domains, C. PCA of GST-like domain Cα coordinates, with first representative structure extraction point indicated, D. PCA of catalytic domain Cα coordinates, with first representative structure extraction point indicated. An additional 200 ns MD simulations was then performed starting from the first representative structure (E-H): E. RMSD progression for the GST-like domain, F. RMSD progression for the catalytic domains, G. PCA of GST-like domain Cα coordinates, with second representative structure extraction point indicated, H. PCA of catalytic domain Cα coordinates, with second representative structure extraction point indicated.

To assess structural stability during simulation, we first calculated RMSD profiles for each replica. All three trajectories showed convergence after ~15 ns, and this initial equilibration period was excluded from subsequent analyses. Domain-wise RMSD revealed that the catalytic region remained stable across simulations, while the GST-like domain exhibited pronounced flexibility and larger structural deviations (Fig. 4A-B). These trends were confirmed by PCA analysis of the Cα atomic positions: the catalytic domain conformed to a tight, reproducible ensemble across replicas, whereas the GST-like domain segregated into multiple distinct conformational clusters, suggesting high mobility and conformational heterogeneity (Fig. 4C-D).

**Fig. 5:**
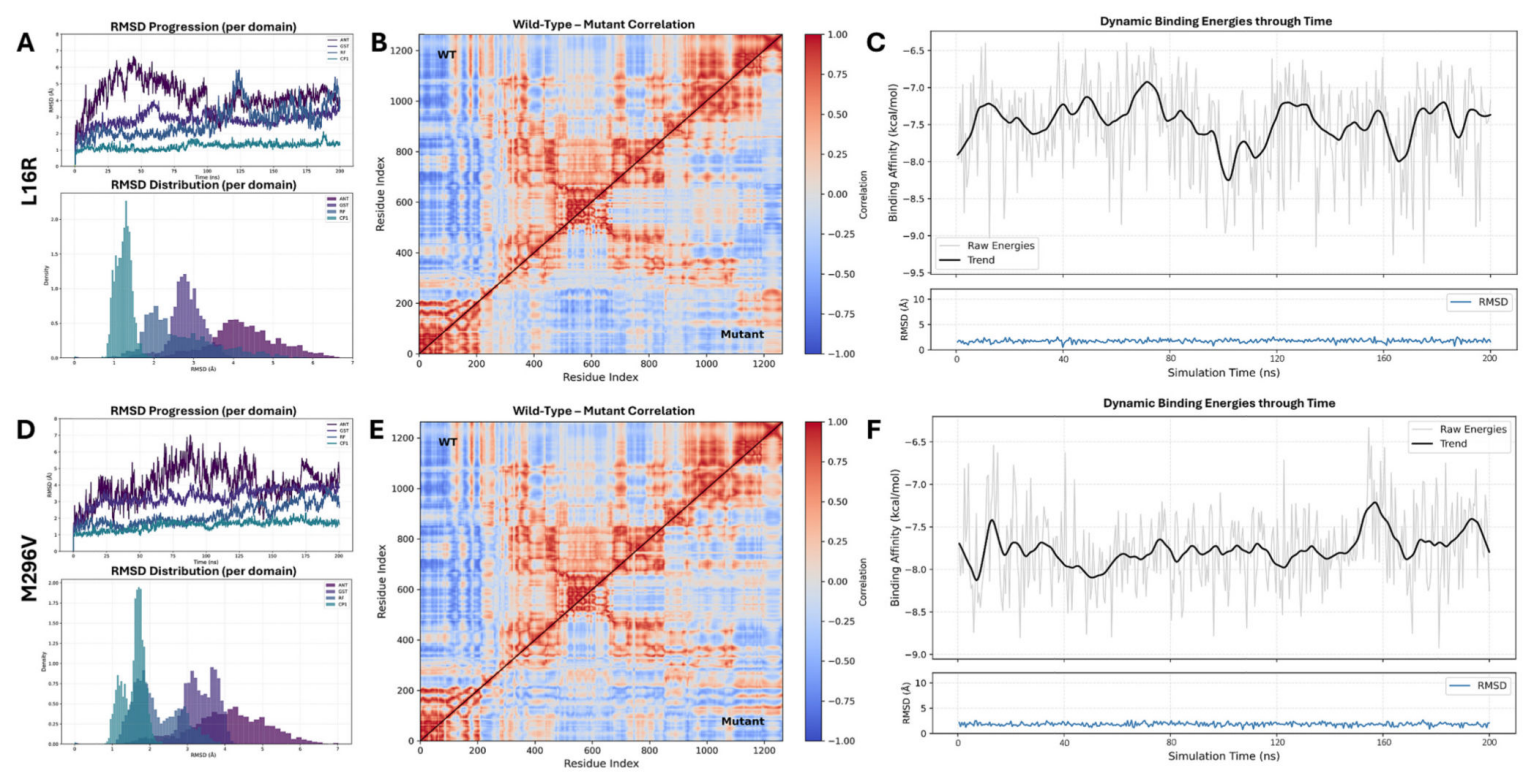
Molecular dynamics analysis of ValRS L16R (A-C) and M296V (D-F) variants. A, D. RMSD progression and distribution per domain: GST-like, CP1, Rossmann fold (RF), and anticodon-binding (ANT). B, E. Dynamic cross-correlation maps comparing residue motion coupling between wild type (upper triangle) and mutant (lower triangle); red (1) indicates correlated motion, blue (–1) indicates anti-correlated motion. C, F. Binding energy profiles over simulation time and RMSD from reference binding pose for each docking pose (see method description in Section 1.4.5 in Supplementary Material): average binding energies were – 7.5 ± 0.5 kcal/mol for L16R and – 7.8 ± 0.4 kcal/mol for M296V. Results from the rest of the studied mutations are presented in Figs. S3-S7 of the Supplementary Material. (For interpretation of the references to colour in this figure legend, the reader is referred to the web version of this article.)

To extract a representative conformation from this dynamic ensemble, we applied RMSD-based clustering (cutoff 1.0 Å) to the combined catalytic domain trajectories, identifying the most populated structural cluster. The centroid of this cluster was selected as a first representative structure and subjected to a second 200 ns MD simulation for further refinement. This additional trajectory confirmed the reproducibility of our observations: the catalytic domain maintained its stability post-equilibration, while the GST domain continued to sample a broader conformational space (Fig. 5A-B). PCA of the refined simulation supported this divergence, with the catalytic domain now forming a unified conformational basin, and the GST domain resolving into three discrete structural clusters (Fig. 5C-D). From these, we selected the centroid of the most populated GST cluster to serve as the final, dynamically equilibrated reference model for all downstream mutant analyses.

Using this refined structure as a biologically grounded template, we modeled each disease-associated mutation and carried out further individual 200 ns MD simulations for each variant alongside the wild-type control. As in the initial refinement, most trajectories stabilized within the first 15 ns, which were excluded from comparative analyses to ensure consistent sampling of post-equilibrated dynamics.

### 3.5 Functional interpretation of ValRS variants using MD simulations

Variants located within both the GST-like domain and the main catalytic domain were analyzed to identify structural perturbations, alterations in dynamic residue correlations, and potential impacts on ligand and tRNA binding. Fig. 5 shows the MD-derived structural analysis for two representative variants: L16R (mutationed GST-like domain) and M296V (mutationed catalytic region). Corresponding analyses for the remaining variants are presented in Figs. S3-S9, and complete trajectory metrics are provided in Supplementary Tables S4-S12.

Substitution of leucine at position 16 with arginine (L16R) within the GST-like domain showed comparable overall stability to the wild-type protein over 200 ns of MD simulation (Fig. 5A). Both the GST-like domain (mutant: 2.8 ± 0.4 Å, wild type: 2.9 ± 0.6 Å) and the catalytic region (mutant: 3.7 ± 1.5 Å, wild type: 2.9 ± 1.0 Å) exhibited similar RMSD values. Individual domain analysis in the catalytic region revealed similar behavior for the Rossmann fold (2.7 ± 0.9 Å *vs*. 2.4 ± 0.6 Å), anticodon-binding domain (4.3 ± 0.9 Å *vs*. 4.2 ± 1.7 Å) and CP1 editing domain (1.2 ± 0.2 Å for both phenotypes) (Table S14). However, cross-correlation analysis revealed slightly altered movement patterns within the aminoacylation and anticodon-interacting regions, despite the L16R substitution being located distant from the catalytic core (Fig. 5B).

The long-range influences in the anticodon-interacting regions are suggested also by the amino acid communication network observation, where more specifically, one residue Pro1232 (ΔBC = –0.0028) important for tRNA binding appeared as more important communication hub for the mutated protein network than in the wild type. At the same time, the same Proline residue (ΔRMSF: +2.1 Å) found to fluctuate less than in the wild type (Table S6). In addition, the ATP-valyl binding energy was slightly negatively affected (–7.5 ± 0.6 kcal/mol in the mutant over – 7.9 ± 0.5 in the wild type) as suggested by the dynamic binding analysis (Fig. 5C).

The Methionine to Valine substitution in position 296 (M296V) is situated adjacent to the catalytic domain and in close proximity to the aminoacylation active site. During the MD simulation the observed structural fluctuation of the GST-like domain (RMSD: 3.3 ± 0.5 Å) and the catalytic region (2.8 ± 0.5 Å) remained unchanged from the wild type (GST-like: 2.9 ± 0.6 Å, catalytic: 2.9 ± 1.0 Å) (Fig. 5D). More specifically for the catalytic region: anticodon-binding domain (mutant: 4.1 ± 1.0 Å, wild type: 4.2 ± 1.7), CP1 editing domain (mutant: 1.6 ± 0.3 Å, wild type: 1.6 ± 0.3 Å) and Rossmann fold domain (mutant: 2.2 ± 0.6 Å, wild type: 2.4 ± 0.6 Å) (Table S14). Finally, dynamic cross-correlation analysis suggested a slightly altered profile particularly for the catalytic core (Fig. 5E).

The MD simulation also suggested deviation in the communication network among the residues from the wild type, promoted by the mutated phenotype (Table S8). Among which we focused on residue Met583 (ΔBC = – 0.0004) important in the threonine selection mechanism catalyzed by the CP1 domain. The negative change in its communication role here, suggests increased hub importance in the mutant phenotype over the wild type. Moreover, measures of residue fluctuations with RMSF revealed Pro1232 (+2.8 Å) and Val1235 (+2.6 Å), two residues important for the tRNA binding, fluctuated again less in the mutant than in the wild type. No residues important for ATP-valyl ligand binding seemed to be impacted by the alteration, as its shown also by the docking energy progression (Fig. 5F) with an average of –7.8 ± 0.4 kcal/mol over the –7.9 ± 0.5 kcal/mol observed in the wild type.

The rest variants studied can be found in section 2.2 of the Supplementary Material.

## 4 Discussion

We report on the molecular and phenotypic spectrum of 13 individuals with biallelic variants in *VARS1*, including the identification of ten novel variants and two cases presenting with mild typical presentation, providing further insight into the genotype–phenotype correlations in NDMSCA. Moreover, this study reports, for the first time, individuals with biallelic *VARS1* variants in French, Brazilian, and Cypriot populations, broadening the global scope of the disorder.

Although the clinical features of patients reported in our study are broadly consistent with previously reported NDMSCA cases, there was notable variability in disease severity, both within our cohort in this study and in comparison with published cases. Progressive cortical atrophy, considered a hallmark of NDMSCA, was notably absent in several of our cases. This further supports previous studies [12,14] suggesting that cortical atrophy is not a distinguishing feature of NDMSCA.

In addition, we observed a milder and non-progressive clinical presentation of NDMSCA in two siblings (Subjects 8 and 9). Notably, both displayed non-progressive microcephaly, later- onset seizures that typically described, mild-to-moderate delayed psychomotor delay and intellectual disability, preserved speech, independent ambulation, and no evidence of cerebellar atrophy or white matter loss. These key phenotypes differ from the expected progressive neurological decline in NDMSCA and challenge the current understanding of the progressive nature of this condition. This observation also provides valuable information for clinicians when diagnosing patients that exhibits phenotypes resembling NDMSCA but as a milder clinical presentation.

Variant-level comparisons also reinforced this phenotypic heterogeneity. The F1072L variant, observed in both our study and in Okur et al. (2018) study as a compound heterozygous state, illustrates this variability. Okur et al. described two prematurely born male siblings, aged 10 and 15, with intellectual disability, developmental delay, severe speech impairment, and microcephaly, though both of our patients who carry the F1072L variant had a relatively milder clinical presentation. This highlights the wide phenotypic spectrum associated with *VARS1* variants [13].

Of note, the R404W variant, previously reported by Siekierska et al. (2019) in association with premature birth and IUGR, was identified in two of our patients [12]. Subjects 6 and 7, had this variant in compound heterozygosity, but were presented with phenotypes similar to Sierkierska’s cases, both in severity and characteristics. Interestingly, we also identified a homozygous variant in subject 2 that affects the same position but the amino acid changes to a glutamine instead of a tryptophan. Subject 2 exhibits more severe clinical features compared to both subjects 6 and 7 as well as the two patients [12]. This suggests that the specific allelic combination and zygosity play a crucial role in shaping the clinical outcome and highlights a broader neurological spectrum for this specific homozygous mutation.

Truncating variants were not observed in a homozygous state and were only found in compound heterozygosity in both our cohort (W790* in family 9 and R442* in family 10) and in previous studies [10,12]. This finding is consistent with the hypothesis that complete loss of VARS1 function is likely incompatible with life. Indeed, Siekierska et al. showed *VARS* knockout zebrafish larvae exhibit premature death within 8 to 12 days post fertilization [12] and no individual with homozygous truncating variants was observed in gnomAD. While no protein prediction modeling was investigated for the identified truncated variants, Friedman et al. had shown protein expression was diminished in the patient of the R442* variant [11].

A cluster of novel *VARS1* variants was identified within the catalytic part of the protein critical for amino acid activation and tRNA charging. This included a likely loss-of-function stop codon variant (W790*), several missense variants (M296V, R404Q, H566Y, R665W, S866C and D920N) with potential functional consequences and a synonymous variant (L631=) whose impact is likely minimal. MD simulations of the M296V and D920N variants revealed behavioral deviations from the wild-type protein, specifically affecting key residues (Met583, Asp584, Pro1232, Val1235 and Lys1242) (Table S5) located within the CP1 editing and anticodon-binding domains. Moreover, the phenotype observed for the R404Q, R665W, H566Y and S866C variants was also characterized by structural shifts in those key tRNA-binding residues (Pro1232, Val1235 and Lys1242), suggesting an potential effect in tRNA binding alone. On the contrary, no significant changes were observed in the valyl-ATP-binding ability of any of those variants. However, a limitation to be addressed here is that while S866C (KMSKS motif) showed no significant change in thermodynamic affinity of the substrate, it may impact the kinetic profile of substrate binding. Our simulations focus on the bound state and thus, changes in the rates of substrate entry or exit remain a potential functional effect that was not captured in this study.

Furthermore, several missense variants were also identified in the anticodon-binding domain (R1058Q, T1068M, F1072L), a region critical for tRNA binding. These variants, along with previously reported pathogenic mutations in this domain, highlight the importance of this region for VARS1 function and suggest that alterations here, even seemingly conservative substitutions, can disrupt tRNA recognition and interaction [10].

The L16R and P51S variants, located adjacent to the GST-like domain, were absent or reported only rarely in public reference databases (EXAC, gnomAD). Although this domain is not directly involved in catalysis, it plays a critical structural role in mediating protein-protein interactions and maintaining spatial organization of the adjacent domains. MD simulations of both variants suggest they induce functionally relevant effects on domains involved in tRNA recognition and binding. Specifically, these mutations exert long-range influence on critical residues such as Pro1232. Additionally, the L16R variant appears to slightly enhance the binding affinity of the valyl-ATP substrate, likely by promoting structural rearrangements within the distal binding pocket. In contrast, while P51S does not affect valyl-ATP binding, it exhibited alterations in the CP1 editing domain by modifying the conformational dynamics of residues Met583 and Asp584.

Despite the comprehensive clinical and genetic analyses presented in this study, several limitations should be acknowledged. Functional validation of the identified *VARS1* variants was limited to *in silico* structural modeling and MD simulations; no *in vitro* or *in vivo* assays were performed to directly assess the impact of these variants on protein function or tRNA aminoacylation activity. The relatively small sample size restrict the generalizability of our findings. Finally, although exome sequencing was utilized, other potential genetic or epigenetic modifiers may have been missed, which could influence phenotypic expression. Future studies with larger cohorts, extended clinical follow-up, and experimental validation are needed to fully elucidate the pathogenic mechanisms of *VARS1*-related disorders.

Despite these limitations, the present study provides valuable and novel contributions to the understanding of *VARS1*-associated neuro-developmental disorders. By integrating detailed clinical characterization with advanced *in silico* structural analyses, we provide a robust framework for interpreting the pathogenicity of novel variants in established genetic disorders. Importantly, the recognition of milder, non-progressive phenotypes expands the known clinical spectrum, challenges the assumption of uniformly progressive disease and has direct implications for refining diagnosis, genetic counseling, and future therapeutic exploration.

To conclude, we identified and characterized ten novel and six previously reported biallelic variants in *VARS1* across thirteen individuals from ten unrelated families, thereby markedly expanding both the genotypic and phenotypic spectrum of *VARS1*-associated neuro-developmental disorder. Our combined clinical, molecular, and *in silico* structural analyses, including MD simulations, provide compelling evidence that these variants, particularly those within the aminoacylation and anticodon-binding domains, alter local protein stability, domain communication, or tRNA interaction in ways consistent with disease causation. Importantly, our findings challenge the prevailing assumption that neurodevelopmental disorder invariably follows a progressive neurological course, as we observed non-progressive, milder phenotypes in some individuals.

By integrating high-resolution computational modeling with detailed phenotypic data, we deliver new mechanistic insights into how distinct *VARS1* variants may perturb enzymatic fidelity or interdomain coordination without necessarily abolishing catalytic activity. These insights not only refine the genotype–phenotype correlations but also have direct implications for clinical diagnosis, prognostic counseling, and future functional studies aimed at therapeutic development.

## Corrigendum to “Novel VARS1 variants define new clinical and molecular subtypes of a rare neurodevelopmental syndrome” [Biochim. Biophys. Acta Mol. Basis Dis. 1872 (2026)/168184]

The authors regret that in the original article, the name of author 7 was incorrectly spelled as ‘Svetlena Gorokhova’ The correct spelling is ‘Svetlana Gorokhova’.

The authors apologize for this error and state that this does not change the scientific conclusions of the article in any way.

https://doi.org/10.1016/j.bbadis.2026.168222

## Supplementary Material

Supplementary data to this article can be found online at https://doi.org/10.1016/j.bbadis.2026.168184.

Supplementary Material

Supplementary Table 1

Supplementary Table 2

## Figures and Tables

**Table 1:** Residue conservation from ConSurf multiple sequence analysis (MSA).

Residue	Conservation score	Confidence interval	MSA data	Residue variety
Leu16	**-0.467**	– 0.915, – 0.222	17/500	L 94%, A 5%
Pro51	**0.639**	– 0.319, 1.390	5/500	P 60%, H 20%, N 20%
Met296	–**0.153**	– 0.319, – 0.110	242/500	L 39%, M 32%, F 10%, Y 3%, A-H 2%, S-I-N-T 1%
Arg404	–**1.021**	– 1.081, – 0.988	496/500	R 92%, K 4%
His566	–**1.057**	– 1.108, – 1.022	500/500	L 52%, H 42%, V 2%
Arg665	–**1.117**	– 1.155, – 1.108	500/500	R 90%, K 9%, S < 1%
Ser866	–**1.150**	– 1.175, – 1.132	499/500	S 96%, T 2%
Asp920	–**1.214**	– 1.226, – 1.209	500/500	D 99%, H < 1%

## Data Availability

Data will be made available on request.
