# Supplementary material for: Novel VARS1 variants define new clinical and molecular subtypes of a rare neurodevelopmental syndrome
Source: Biochim Biophys Acta Mol Basis Dis. Author manuscript; Available in PMC 2026 Aug 12. (PMC7619359; doi:10.1016/j.bbadis.2026.168184)
Supplement: Supplementary file 1 [file EMS212799-supplement-Supplementary_Material.docx]

**Supplementary Material**

| **Table S3:** Experimentally-characterized pathogenic and likely pathogenic VARS1 variants associated with neurodevelopmental disorder with microcephaly, seizures, and cortical atrophy | |
| --- | --- |
| **Alteration** | **Reported Effect** |
| **A22D** [1] | Missense; compound het with F1072L in siblings with ID and microcephaly; VUS (no functional studies performed) |
| **L78Argfs*35** [2] | Null allele; Nonsense-Mediated Decay (NMD) confirmed; causes ~50% reduction in VARS protein; compound het with R942Q |
| **Q400P** [2] | Missense; predicted to weaken local interactions; failed zebrafish rescue; loss of function mutation |
| **R404W** [2] | Homozygous in multiple families; ~25% residual aminoacylation activity; likely impairs folding/function |
| **L434V** [2] | Hypomorphic; yeast complementation positive (near-normal growth); ~50% reduced aminoacylation in patient cells; compound het with G822S |
| **R442Q** [2] | Yeast complementation positive (~WT activity); mild/hypomorphic allele; causes disease only in trans with severe allele |
| **P661T** [3] | Homozygous; reduces aminoacylation activity in patient cells; partial loss of function |
| **A692P** [3] | Compound het with null allele; reduced aminoacylation in patient cells; partial loss of function |
| **G822S** [2] | Functional null; abolished yeast growth (no complementation); no tRNA charging |
| **L885F** [4] | Homozygous; yeast complementation positive (near-normal growth); mild activity reduction |
| **R942Q** [2] | Directly contacts tRNA; predicted to impair tRNA binding; partial zebrafish rescue; hypomorphic |
| **R947H** [3] | Homozygous; conserved Arg; predicted to disrupt tRNA recognition |
| **R1058Q** [2,4] | Homozygous; failed zebrafish rescue; makes few stabilizing contacts (predicted milder structural effect but functional loss of function) |
| **T1068M** [5] | Homozygous; loses stabilizing interhelical contact with S1061; clusters in 15-aa disease hotspot |
| **F1072L** [1] | Compound het with A22D; predicted to destabilize tRNA binding; Variant of uncertain significance (no functional studies) |
| **R1119C** [3] | Recurrent founder mutation; homozygous; disrupts conserved Arg and local helix structure; impairs anticodon recognition |

| **Table S4:** Conservation of KMSKS Motif from ConSurf Multiple Sequence Analysis (MSA) | | | | |
| --- | --- | --- | --- | --- |
| **Residue** | **Conservation Score** | **Confidence Interval** | **MSA data** | **Residue Variety** |
| Lys862 | **-1.171** | -1.209, -1.155 | 500/500 | K 98% |
| Met863 | **-1.209** | -1.222, -1.209 | 500/500 | M 99% |
| Ser864 | **-1.197** | -1.222, -1.194 | 500/500 | S 99% |
| Lys865 | **-1.212** | -1.226, -1.209 | 499/500 | K 99% |
| Ser866 | **-1.150** | -1.175, -1.132 | 499/500 | S 96% |

| **Table S5**: Structurally important residues in the ValRS protein and their alignment between the human (UniProt: P26640) and a reference bacterial ValRS (UniProt: P96142). | | | | | |
| --- | --- | --- | --- | --- | --- |
| Valine binding | | tRNA binding | | Threonine selection | |
| ***T. thermophilus*** | ***H. sapiens*** | ***T. thermophilus*** | ***H. sapiens*** | ***T. thermophilus*** | ***H. sapiens*** |
| Pro41 | Asp342 | Arg570 | Asn941 | Arg216 | Arg537 |
| Pro42 | Pro343 | Arg576 | Arg947 | Lys262 | Met583 |
| Asn44 | Asn345 | Asn580 | Asn951 | Asp263 | Asp584 |
| His50 | His351 | Lys581 | Lys952 | Phe264 | Phe585 |
| His53 | His354 | Asn584 | Asn955 | Gly265 | Gly586 |
| Asp81 | Asp382 | Arg587 | Lys958 | Thr266 | Thr587 |
| Trp456 | Trp790 | Phe588 | Phe959 | Gly267 | Gly588 |
| Thr487 | Thr821 | Cys646 | Cys1023 | Ala268 | Ala589 |
| Gly488 | Gly822 | Leu650 | Leu1027 | Lys270 | Lys591 |
| Asp490 | Asp824 | Glu651 | Glu1028 | Thr272 | Thr593 |
| Ile491 | Ile825 | Leu815 | Gln1214 | Asp276 | Asp597 |
| Trp495 | Trp829 | Arg818 | Arg1217 | Asp279 | Asp600 |
| His518 | His852 | Pro833 | Pro1232 |  |  |
| Leu520 | Ile854 | Val836 | Val1235 |  |  |
| Val521 | Val855 | Arg843 | Lys1242 |  |  |
| Met529 | Met863 | Asn847 | Thr1246 |  |  |

| **Table S6**: Top 20 residues with increased and top 20 residues decreased communication hub importance (Betweenness Centrality) and structural flexibility (RMSF) in the ValRS **L16R** variant compared to the wild-type. | | | | | | | | |
| --- | --- | --- | --- | --- | --- | --- | --- | --- |
| **Betweenness Centrality** | | | **RMSF (Å)**  (Catalytic) | | | **RMSF (Å)**  (GST-like domain) | | |
| **Position** | **ΔBetweenness**  (WT-MUT) | **Importance** | **Position** | **ΔRMSF**  (WT-MUT) | **Importance** | **Position** | **ΔRMSF**  (WT-MUT) | **Importance** |
| 487 | -0,0065 | - | 282 | -0,6 | - | 121 | -2,8 | - |
| 1233 | -0,0044 | - | 1082 | -0,6 | - | 93 | -2,3 | - |
| 1232 | -0,0028 | RNABind | 932 | -0,6 | - | 118 | -2,3 | - |
| 224 | -0,002 | - | 1195 | -0,5 | - | 94 | -2,3 | - |
| 1231 | -0,0018 | - | 1184 | -0,4 | - | 119 | -2,0 | - |
| 154 | -0,001 | - | 1194 | -0,4 | - | 97 | -2,0 | - |
| 263 | -0,0009 | - | 860 | -0,4 | - | 51 | -2,0 | - |
| 404 | -0,0006 | - | 1080 | -0,3 | - | 95 | -1,9 | - |
| 28 | -0,0005 | - | 859 | -0,3 | - | 50 | -1,9 | - |
| 254 | -0,0004 | - | 717 | -0,3 | - | 47 | -1,8 | - |
| 1228 | -0,0003 | - | 1081 | -0,3 | - | 9 | -1,8 | - |
| 895 | -0,0003 | - | 861 | -0,3 | - | 98 | -1,8 | - |
| 118 | -0,0002 | - | 858 | -0,3 | - | 115 | -1,8 | - |
| 1080 | -0,0002 | - | 1083 | -0,2 | - | 122 | -1,8 | - |
| 741 | -0,0002 | - | 862 | -0,2 | - | 52 | -1,7 | - |
| 197 | -0,0002 | - | 284 | -0,2 | - | 101 | -1,7 | - |
| 1229 | -0,0002 | - | 935 | -0,2 | - | 48 | -1,7 | - |
| 1123 | -0,0001 | - | 857 | -0,1 | - | 56 | -1,7 | - |
| 896 | -0,0001 | - | 348 | -0,1 | - | 11 | -1,7 | - |
| 978 | -0,0001 | - | 283 | -0,1 | - | 96 | -1,7 | - |
| 325 | 0,0003 | - | 330 | 3,1 | - | 218 | 4,0 | - |
| 1083 | 0,0003 | - | 331 | 3,1 | - | 217 | 3,0 | - |
| 330 | 0,0004 | - | 329 | 2,6 | - | 87 | 1,8 | - |
| 147 | 0,0005 | - | 561 | 2,6 | - | 216 | 1,8 | - |
| 120 | 0,0006 | - | 557 | 2,6 | - | 88 | 1,7 | - |
| 48 | 0,0006 | - | 558 | 2,6 | - | 30 | 1,3 | - |
| 326 | 0,0006 | - | 1234 | 2,5 | - | 31 | 0,9 | - |
| 1019 | 0,0006 | - | 560 | 2,5 | - | 215 | 0,9 | - |
| 258 | 0,0009 | - | 554 | 2,5 | - | 89 | 0,7 | - |
| 259 | 0,0012 | - | 524 | 2,4 | - | 125 | 0,6 | - |
| 579 | 0,0013 | - | 562 | 2,4 | - | 86 | 0,6 | - |
| 297 | 0,0015 | - | 526 | 2,4 | - | 34 | 0,5 | - |
| 266 | 0,0026 | - | 1233 | 2,3 | - | 33 | 0,5 | - |
| 1167 | 0,0028 | - | 488 | 2,3 | - | 29 | 0,3 | - |
| 1168 | 0,0038 | - | 525 | 2,3 | - | 126 | 0,2 | - |
| 274 | 0,004 | - | 559 | 2,3 | - | 136 | 0,2 | - |
| 219 | 0,007 | - | 523 | 2,2 | - | 137 | 0,1 | - |
| 212 | 0,0076 | - | 332 | 2,2 | - | 35 | 0,1 | - |
| 228 | 0,0096 | - | 527 | 2,2 | - | 214 | 0,0 | - |
| 1182 | 0,0165 | - | 1232 | 2,1 | RNABind | 38 | 0,0 | - |

| **Table S7**: Top 20 residues with increased and top 20 residues decreased communication hub importance (Betweenness Centrality) and structural flexibility (RMSF) in the ValRS **P51S** variant compared to the wild-type. | | | | | | | | |
| --- | --- | --- | --- | --- | --- | --- | --- | --- |
| **Betweenness Centrality** | | | **RMSF (Å)**  (Catalytic) | | | **RMSF (Å)**  (GST-like domain) | | |
| **Position** | **ΔBetweenness**  (WT-MUT) | **Importance** | **Position** | **ΔRMSF**  (WT-MUT) | **Importance** | **Position** | **ΔRMSF**  (WT-MUT) | **Importance** |
| 487 | -0,0147 | - | 895 | -1,7 | - | 36 | -2,6 | - |
| 263 | -0,0098 | - | 897 | -1,7 | - | 37 | -2,5 | - |
| 907 | -0,0092 | - | 898 | -1,6 | - | 38 | -1,9 | - |
| 225 | -0,0074 | - | 894 | -1,6 | - | 118 | -1,9 | - |
| 933 | -0,0024 | - | 896 | -1,6 | - | 90 | -1,6 | - |
| 86 | -0,0023 | - | 893 | -1,5 | - | 35 | -1,5 | - |
| 1233 | -0,0018 | - | 892 | -1,4 | - | 32 | -1,5 | - |
| 584 | -0,0014 | ThrBind | 901 | -1,4 | - | 89 | -1,4 | - |
| 404 | -0,0011 | - | 900 | -1,3 | - | 91 | -1,4 | - |
| 501 | -0,001 | - | 899 | -1,3 | - | 31 | -1,4 | - |
| 583 | -0,0004 | ThrBind | 891 | -1,2 | - | 121 | -1,1 | - |
| 1036 | -0,0004 | - | 282 | -1,2 | - | 92 | -1,0 | - |
| 154 | -0,0003 | - | 890 | -1,2 | - | 34 | -1,0 | - |
| 257 | -0,0003 | - | 902 | -1,2 | - | 117 | -1,0 | - |
| 282 | -0,0003 | - | 904 | -1,1 | - | 87 | -1,0 | - |
| 9 | -0,0002 | - | 889 | -1,1 | - | 29 | -0,9 | - |
| 552 | -0,0002 | - | 905 | -1,0 | - | 88 | -0,9 | - |
| 1080 | -0,0002 | - | 903 | -1,0 | - | 30 | -0,9 | - |
| 1002 | -0,0002 | - | 888 | -0,9 | - | 85 | -0,8 | - |
| 1125 | -0,0002 | - | 332 | -0,8 | - | 86 | -0,8 | - |
| 397 | 0,0003 | - | 1232 | 2,9 | RNABind | 126 | 1,7 | - |
| 1168 | 0,0003 | - | 1230 | 2,9 | - | 125 | 1,5 | - |
| 1083 | 0,0004 | - | 1229 | 2,9 | - | 218 | 1,5 | - |
| 330 | 0,0004 | - | 1233 | 2,7 | - | 127 | 1,1 | - |
| 147 | 0,0005 | - | 1231 | 2,7 | - | 124 | 1,1 | - |
| 326 | 0,0006 | - | 1234 | 2,7 | - | 131 | 0,9 | - |
| 1019 | 0,0006 | - | 558 | 2,7 | - | 128 | 0,9 | - |
| 48 | 0,0006 | - | 1235 | 2,6 | RNABind | 65 | 0,8 | - |
| 120 | 0,0007 | - | 557 | 2,6 | - | 217 | 0,7 | - |
| 258 | 0,0009 | - | 1228 | 2,5 | - | 130 | 0,7 | - |
| 259 | 0,0012 | - | 1226 | 2,5 | - | 67 | 0,6 | - |
| 579 | 0,0013 | - | 561 | 2,4 | - | 132 | 0,5 | - |
| 297 | 0,0014 | - | 1227 | 2,4 | - | 82 | 0,5 | - |
| 266 | 0,0026 | - | 560 | 2,4 | - | 129 | 0,5 | - |
| 1167 | 0,0028 | - | 554 | 2,3 | - | 47 | 0,4 | - |
| 274 | 0,004 | - | 1236 | 2,3 | - | 66 | 0,4 | - |
| 219 | 0,007 | - | 524 | 2,2 | - | 64 | 0,4 | - |
| 212 | 0,0076 | - | 559 | 2,2 | - | 11 | 0,4 | - |
| 228 | 0,0097 | - | 488 | 2,2 | - | 216 | 0,4 | - |
| 1182 | 0,0165 | - | 1237 | 2,2 | - | 46 | 0,4 | - |

| **Table S8**: Top 20 residues with increased and top 20 residues decreased communication hub importance (Betweenness Centrality) and structural flexibility (RMSF) in the ValRS **M296V** variant compared to the wild-type. | | | | | | | | |
| --- | --- | --- | --- | --- | --- | --- | --- | --- |
| **Betweenness Centrality** | | | **RMSF (Å)**  (Catalytic) | | | **RMSF (Å)**  (GST-like domain) | | |
| **Position** | **ΔBetweenness**  (WT-MUT) | **Importance** | **Position** | **ΔRMSF**  (WT-MUT) | **Importance** | **Position** | **ΔRMSF**  (WT-MUT) | **Importance** |
| 487 | -0,0434 | - | 284 | -4,9 | - | 91 | -2,0 | - |
| 224 | -0,0086 | - | 282 | -4,8 | - | 92 | -1,6 | - |
| 9 | -0,0029 | - | 283 | -4,6 | - | 129 | -1,1 | - |
| 257 | -0,0024 | - | 285 | -4,3 | - | 124 | -1,0 | - |
| 263 | -0,0019 | - | 748 | -4,2 | - | 51 | -0,8 | - |
| 88 | -0,0014 | - | 747 | -3,7 | - | 125 | -0,8 | - |
| 896 | -0,0008 | - | 287 | -3,2 | - | 50 | -0,7 | - |
| 1233 | -0,0004 | - | 286 | -3,2 | - | 72 | -0,7 | - |
| 486 | -0,0003 | - | 746 | -2,8 | - | 53 | -0,6 | - |
| 1080 | -0,0003 | - | 745 | -2,6 | - | 47 | -0,6 | - |
| 583 | -0,0003 | ThrBind | 288 | -2,5 | - | 55 | -0,6 | - |
| 149 | -0,0003 | - | 293 | -2,4 | - | 123 | -0,6 | - |
| 1231 | -0,0003 | - | 292 | -2,4 | - | 127 | -0,5 | - |
| 55 | -0,0002 | - | 749 | -2,4 | - | 52 | -0,5 | - |
| 1192 | -0,0002 | - | 289 | -1,9 | - | 29 | -0,5 | - |
| 154 | -0,0002 | - | 294 | -1,7 | - | 49 | -0,4 | - |
| 741 | -0,0002 | - | 897 | -1,6 | - | 48 | -0,4 | - |
| 220 | -0,0002 | - | 290 | -1,5 | - | 11 | -0,4 | - |
| 197 | -0,0002 | - | 896 | -1,5 | - | 128 | -0,4 | - |
| 1229 | -0,0002 | - | 895 | -1,5 | - | 126 | -0,4 | - |
| 397 | 0,0003 | - | 1234 | 3,0 | - | 218 | 4,3 | - |
| 579 | 0,0003 | - | 1233 | 2,9 | - | 217 | 3,6 | - |
| 325 | 0,0003 | - | 1232 | 2,8 | RNABind | 216 | 3,3 | - |
| 1083 | 0,0004 | - | 1237 | 2,6 | - | 215 | 2,6 | - |
| 147 | 0,0005 | - | 1235 | 2,6 | RNABind | 214 | 1,2 | - |
| 326 | 0,0006 | - | 1229 | 2,5 | - | 213 | 0,9 | - |
| 1019 | 0,0006 | - | 1231 | 2,4 | - | 89 | 0,8 | - |
| 48 | 0,0006 | - | 1228 | 2,4 | - | 131 | 0,7 | - |
| 120 | 0,0007 | - | 1236 | 2,4 | - | 212 | 0,6 | - |
| 258 | 0,0009 | - | 1230 | 2,4 | - | 88 | 0,5 | - |
| 259 | 0,0012 | - | 1238 | 2,4 | - | 132 | 0,4 | - |
| 297 | 0,0015 | - | 1241 | 2,1 | - | 90 | 0,4 | - |
| 274 | 0,0019 | - | 1240 | 2,1 | - | 66 | 0,2 | - |
| 266 | 0,0023 | - | 1227 | 2,0 | - | 2 | 0,2 | - |
| 1167 | 0,0028 | - | 1226 | 2,0 | - | 206 | 0,1 | - |
| 1168 | 0,0038 | - | 1239 | 1,9 | - | 152 | 0,1 | - |
| 219 | 0,007 | - | 664 | 1,8 | - | 133 | 0,1 | - |
| 212 | 0,0076 | - | 1225 | 1,8 | - | 207 | 0,1 | - |
| 228 | 0,0097 | - | 1244 | 1,8 | - | 43 | 0,1 | - |
| 1182 | 0,0166 | - | 666 | 1,8 | - | 87 | 0,1 | - |

| **Table S9**: Top 20 residues with increased and top 20 residues decreased communication hub importance (Betweenness Centrality) and structural flexibility (RMSF) in the ValRS **R404Q** variant compared to the wild-type. | | | | | | | | |
| --- | --- | --- | --- | --- | --- | --- | --- | --- |
| **Betweenness Centrality** | | | **RMSF (Å)**  (Catalytic) | | | **RMSF (Å)**  (GST-like domain) | | |
| **Position** | **ΔBetweenness**  (WT-MUT) | **Importance** | **Position** | **ΔRMSF**  (WT-MUT) | **Importance** | **Position** | **ΔRMSF**  (WT-MUT) | **Importance** |
| 225 | -0,0031 | - | 332 | -0,9 | - | 216 | -1,1 | - |
| 1233 | -0,0025 | - | 333 | -0,8 | - | 32 | -1,0 | - |
| 9 | -0,0012 | - | 866 | -0,7 | - | 214 | -1,0 | - |
| 1140 | -0,0010 | - | 400 | -0,7 | - | 215 | -0,9 | - |
| 1231 | -0,0010 | - | 406 | -0,7 | - | 91 | -0,7 | - |
| 1128 | -0,0007 | - | 399 | -0,6 | - | 217 | -0,7 | - |
| 88 | -0,0006 | - | 410 | -0,6 | - | 31 | -0,6 | - |
| 263 | -0,0006 | - | 401 | -0,6 | - | 218 | -0,6 | - |
| 154 | -0,0004 | - | 867 | -0,6 | - | 213 | -0,5 | - |
| 1227 | -0,0004 | - | 407 | -0,6 | - | 33 | -0,5 | - |
| 1080 | -0,0004 | - | 408 | -0,6 | - | 34 | -0,4 | - |
| 86 | -0,0003 | - | 411 | -0,6 | - | 35 | -0,4 | - |
| 197 | -0,0003 | - | 398 | -0,5 | - | 125 | -0,3 | - |
| 1166 | -0,0003 | - | 897 | -0,5 | - | 90 | -0,2 | - |
| 294 | -0,0003 | - | 282 | -0,5 | - | 10 | -0,2 | - |
| 230 | -0,0003 | - | 402 | -0,5 | - | 126 | -0,2 | - |
| 404 | -0,0003 | - | 409 | -0,5 | - | 30 | -0,2 | - |
| 1083 | -0,0002 | - | 896 | -0,5 | - | 114 | -0,2 | - |
| 485 | -0,0002 | - | 396 | -0,5 | - | 11 | -0,2 | - |
| 28 | -0,0002 | - | 397 | -0,5 | - | 85 | -0,2 | - |
| 397 | 0,0003 | - | 1234 | 3,7 | - | 122 | 0,5 | - |
| 330 | 0,0004 | - | 1233 | 3,6 | - | 120 | 0,5 | - |
| 48 | 0,0005 | - | 1232 | 3,6 | RNABind | 121 | 0,5 | - |
| 147 | 0,0005 | - | 1229 | 3,3 | - | 123 | 0,5 | - |
| 326 | 0,0006 | - | 1235 | 3,2 | RNABind | 128 | 0,4 | - |
| 1019 | 0,0006 | - | 1230 | 3,1 | - | 50 | 0,3 | - |
| 120 | 0,0006 | - | 1231 | 3,1 | - | 131 | 0,3 | - |
| 258 | 0,0009 | - | 1237 | 3,1 | - | 82 | 0,3 | - |
| 259 | 0,0012 | - | 1228 | 3,0 | - | 84 | 0,3 | - |
| 579 | 0,0013 | - | 1236 | 3,0 | - | 132 | 0,3 | - |
| 297 | 0,0014 | - | 1238 | 2,8 | - | 28 | 0,3 | - |
| 266 | 0,0021 | - | 1227 | 2,5 | - | 119 | 0,3 | - |
| 487 | 0,0027 | - | 1226 | 2,5 | - | 116 | 0,3 | - |
| 1167 | 0,0028 | - | 1241 | 2,5 | - | 65 | 0,2 | - |
| 274 | 0,0035 | - | 1240 | 2,4 | - | 88 | 0,2 | - |
| 1168 | 0,0036 | - | 1239 | 2,3 | - | 66 | 0,2 | - |
| 228 | 0,0046 | - | 1225 | 2,2 | - | 133 | 0,2 | - |
| 219 | 0,0070 | - | 1242 | 2,0 | RNABind | 1 | 0,2 | - |
| 212 | 0,0076 | - | 1222 | 2,0 | - | 2 | 0,2 | - |
| 1182 | 0,0165 | - | 1244 | 1,9 | - | 124 | 0,2 | - |

| **Table S10**: Top 20 residues with increased and top 20 residues decreased communication hub importance (Betweenness Centrality) and structural flexibility (RMSF) in the ValRS **R665W** variant compared to the wild-type. | | | | | | | | |
| --- | --- | --- | --- | --- | --- | --- | --- | --- |
| **Betweenness Centrality** | | | **RMSF (Å)**  (Catalytic) | | | **RMSF (Å)**  (GST-like domain) | | |
| **Position** | **ΔBetweenness**  (WT-MUT) | **Importance** | **Position** | **ΔRMSF**  (WT-MUT) | **Importance** | **Position** | **ΔRMSF**  (WT-MUT) | **Importance** |
| 218 | -0,0428 | - | 859 | -0,6 | - | 118 | -1,3 | - |
| 219 | -0,0389 | - | 1033 | -0,6 | - | 119 | -0,9 | - |
| 487 | -0,0239 | - | 858 | -0,6 | - | 114 | -0,9 | - |
| 224 | -0,0088 | - | 893 | -0,6 | - | 117 | -0,8 | - |
| 853 | -0,0084 | - | 1034 | -0,5 | - | 115 | -0,7 | - |
| 1128 | -0,0026 | - | 584 | -0,5 | ThrBind | 34 | -0,5 | - |
| 263 | -0,0018 | - | 897 | -0,5 | - | 49 | -0,4 | - |
| 88 | -0,0009 | - | 900 | -0,5 | - | 50 | -0,4 | - |
| 1233 | -0,0008 | - | 861 | -0,5 | - | 134 | -0,4 | - |
| 1036 | -0,0006 | - | 896 | -0,5 | - | 35 | -0,4 | - |
| 1125 | -0,0006 | - | 901 | -0,5 | - | 111 | -0,4 | - |
| 404 | -0,0006 | - | 1204 | -0,5 | - | 129 | -0,3 | - |
| 232 | -0,0005 | - | 860 | -0,5 | - | 120 | -0,3 | - |
| 896 | -0,0003 | - | 890 | -0,5 | - | 130 | -0,3 | - |
| 231 | -0,0003 | - | 1205 | -0,5 | - | 33 | -0,3 | - |
| 284 | -0,0003 | - | 904 | -0,5 | - | 10 | -0,3 | - |
| 84 | -0,0002 | - | 895 | -0,5 | - | 51 | -0,3 | - |
| 656 | -0,0002 | - | 898 | -0,5 | - | 53 | -0,2 | - |
| 254 | -0,0002 | - | 1181 | -0,4 | - | 48 | -0,2 | - |
| 583 | -0,0002 | ThrBind | 899 | -0,4 | - | 133 | -0,2 | - |
| 397 | 0,0003 | - | 1234 | 4,9 | - | 218 | 4,6 | - |
| 30 | 0,0003 | - | 1233 | 4,7 | - | 217 | 4,2 | - |
| 933 | 0,0003 | - | 1232 | 4,7 | RNABind | 216 | 3,7 | - |
| 1083 | 0,0004 | - | 1235 | 4,2 | RNABind | 215 | 2,7 | - |
| 330 | 0,0004 | - | 1229 | 4,0 | - | 88 | 2,6 | - |
| 147 | 0,0005 | - | 1231 | 4,0 | - | 89 | 2,5 | - |
| 326 | 0,0006 | - | 1230 | 4,0 | - | 87 | 1,8 | - |
| 1019 | 0,0006 | - | 1237 | 3,9 | - | 90 | 1,7 | - |
| 48 | 0,0006 | - | 1236 | 3,7 | - | 214 | 1,5 | - |
| 274 | 0,0008 | - | 1238 | 3,6 | - | 91 | 1,1 | - |
| 258 | 0,0009 | - | 1228 | 3,6 | - | 213 | 0,9 | - |
| 259 | 0,0012 | - | 1226 | 3,0 | - | 86 | 0,8 | - |
| 579 | 0,0013 | - | 1227 | 3,0 | - | 122 | 0,8 | - |
| 266 | 0,0014 | - | 1241 | 2,9 | - | 212 | 0,7 | - |
| 297 | 0,0014 | - | 1239 | 2,8 | - | 92 | 0,7 | - |
| 1167 | 0,0028 | - | 1240 | 2,7 | - | 82 | 0,6 | - |
| 1168 | 0,0038 | - | 1242 | 2,2 | RNABind | 30 | 0,6 | - |
| 212 | 0,0076 | - | 331 | 2,2 | - | 121 | 0,6 | - |
| 228 | 0,0095 | - | 330 | 2,1 | - | 67 | 0,5 | - |
| 1182 | 0,0165 | - | 1244 | 1,9 | - | 1 | 0,4 | - |

| **Table S11**: Top 20 residues with increased and top 20 residues decreased communication hub importance (Betweenness Centrality) and structural flexibility (RMSF) in the ValRS **S866C** variant compared to the wild-type. | | | | | | | | |
| --- | --- | --- | --- | --- | --- | --- | --- | --- |
| **Betweenness Centrality** | | | **RMSF (Å)**  (Catalytic) | | | **RMSF (Å)**  (GST-like domain) | | |
| **Position** | **ΔBetweenness**  (WT-MUT) | **Importance** | **Position** | **ΔRMSF**  (WT-MUT) | **Importance** | **Position** | **ΔRMSF**  (WT-MUT) | **Importance** |
| 487 | -0,0345 | - | 746 | -2,8 | - | 90 | -1,5 | - |
| 1168 | -0,0092 | - | 747 | -2,8 | - | 89 | -1,3 | - |
| 1231 | -0,0054 | - | 743 | -2,7 | - | 91 | -1,0 | - |
| 224 | -0,0045 | - | 744 | -2,6 | - | 88 | -0,6 | - |
| 1228 | -0,0034 | - | 329 | -2,5 | - | 92 | -0,6 | - |
| 1229 | -0,0029 | - | 742 | -2,5 | - | 86 | -0,5 | - |
| 1233 | -0,0029 | - | 748 | -2,5 | - | 64 | -0,4 | - |
| 1232 | -0,002 | RNABind | 745 | -2,4 | - | 63 | -0,4 | - |
| 122 | -0,0019 | - | 328 | -2,3 | - | 66 | -0,4 | - |
| 271 | -0,0017 | - | 741 | -2,1 | - | 37 | -0,2 | - |
| 84 | -0,0016 | - | 330 | -1,5 | - | 207 | -0,2 | - |
| 579 | -0,0014 | - | 331 | -1,5 | - | 1 | -0,1 | - |
| 205 | -0,0012 | - | 749 | -1,5 | - | 62 | -0,1 | - |
| 9 | -0,0011 | - | 333 | -1,3 | - | 114 | -0,1 | - |
| 257 | -0,0006 | - | 398 | -1,2 | - | 71 | -0,1 | - |
| 1138 | -0,0006 | - | 401 | -1,2 | - | 87 | -0,1 | - |
| 55 | -0,0005 | - | 897 | -1,1 | - | 73 | -0,1 | - |
| 197 | -0,0004 | - | 327 | -1,1 | - | 85 | -0,1 | - |
| 495 | -0,0003 | - | 898 | -1,1 | - | 67 | -0,1 | - |
| 254 | -0,0003 | - | 859 | -1,1 | - | 11 | -0,1 | - |
| 397 | 0,0003 | - | 554 | 2,2 | - | 218 | 4,3 | - |
| 30 | 0,0003 | - | 557 | 2,2 | - | 217 | 3,6 | - |
| 933 | 0,0003 | - | 558 | 2,0 | - | 216 | 2,9 | - |
| 325 | 0,0003 | - | 555 | 1,9 | - | 215 | 2,3 | - |
| 1083 | 0,0004 | - | 561 | 1,9 | - | 124 | 1,7 | - |
| 48 | 0,0004 | - | 553 | 1,9 | - | 123 | 1,5 | - |
| 147 | 0,0005 | - | 556 | 1,8 | - | 125 | 1,5 | - |
| 326 | 0,0006 | - | 528 | 1,8 | - | 126 | 1,2 | - |
| 1019 | 0,0006 | - | 527 | 1,8 | - | 33 | 1,2 | - |
| 120 | 0,0006 | - | 526 | 1,8 | - | 214 | 1,2 | - |
| 258 | 0,0009 | - | 562 | 1,7 | - | 122 | 1,0 | - |
| 259 | 0,0012 | - | 559 | 1,7 | - | 83 | 0,8 | - |
| 297 | 0,0014 | - | 524 | 1,7 | - | 34 | 0,8 | - |
| 274 | 0,0021 | - | 551 | 1,7 | - | 131 | 0,7 | - |
| 266 | 0,0025 | - | 552 | 1,7 | - | 52 | 0,7 | - |
| 1167 | 0,0028 | - | 525 | 1,7 | - | 127 | 0,7 | - |
| 219 | 0,007 | - | 529 | 1,7 | - | 82 | 0,7 | - |
| 212 | 0,0076 | - | 523 | 1,7 | - | 213 | 0,7 | - |
| 228 | 0,0096 | - | 646 | 1,6 | - | 121 | 0,7 | - |
| 1182 | 0,0165 | - | 647 | 1,6 | - | 50 | 0,6 | - |

| **Table S12**: Top 20 residues with increased and top 20 residues decreased communication hub importance (Betweenness Centrality) and structural flexibility (RMSF) in the ValRS **D920N** variant compared to the wild-type. | | | | | | | | |
| --- | --- | --- | --- | --- | --- | --- | --- | --- |
| **Betweenness Centrality** | | | **RMSF (Å)**  (Catalytic) | | | **RMSF (Å)**  (GST-like domain) | | |
| **Position** | **ΔBetweenness**  (WT-MUT) | **Importance** | **Position** | **ΔRMSF**  (WT-MUT) | **Importance** | **Position** | **ΔRMSF**  (WT-MUT) | **Importance** |
| 487 | -0,0132 | - | 282 | -3,2 | - | 85 | -0,9 | - |
| 224 | -0,0058 | - | 283 | -2,2 | - | 86 | -0,6 | - |
| 1233 | -0,0042 | - | 284 | -1,8 | - | 84 | -0,5 | - |
| 1232 | -0,0035 | RNABind | 746 | -1,6 | - | 64 | -0,3 | - |
| 486 | -0,0025 | - | 747 | -1,6 | - | 29 | -0,3 | - |
| 1231 | -0,0021 | - | 748 | -1,4 | - | 73 | -0,3 | - |
| 262 | -0,0013 | - | 745 | -1,2 | - | 114 | -0,3 | - |
| 263 | -0,0012 | - | 285 | -0,9 | - | 87 | -0,3 | - |
| 9 | -0,0007 | - | 749 | -0,6 | - | 36 | -0,3 | - |
| 294 | -0,0006 | - | 508 | -0,6 | - | 63 | -0,3 | - |
| 743 | -0,0005 | - | 507 | -0,6 | - | 37 | -0,3 | - |
| 282 | -0,0004 | - | 509 | -0,5 | - | 28 | -0,2 | - |
| 584 | -0,0004 | ThrBind | 665 | -0,5 | - | 71 | -0,2 | - |
| 197 | -0,0004 | - | 333 | -0,5 | - | 118 | -0,2 | - |
| 254 | -0,0004 | - | 635 | -0,5 | - | 12 | -0,2 | - |
| 1229 | -0,0003 | - | 287 | -0,4 | - | 13 | -0,2 | - |
| 28 | -0,0003 | - | 668 | -0,4 | - | 11 | -0,2 | - |
| 1080 | -0,0003 | - | 744 | -0,4 | - | 15 | -0,2 | - |
| 55 | -0,0003 | - | 638 | -0,4 | - | 121 | -0,2 | - |
| 404 | -0,0003 | - | 666 | -0,4 | - | 9 | -0,2 | - |
| 325 | 0,0003 | - | 1234 | 3,7 | - | 218 | 2,2 | - |
| 397 | 0,0003 | - | 1233 | 3,6 | - | 217 | 2,1 | - |
| 1083 | 0,0004 | - | 1232 | 3,4 | RNABind | 216 | 1,9 | - |
| 147 | 0,0004 | - | 1237 | 3,2 | - | 89 | 1,4 | - |
| 48 | 0,0005 | - | 1235 | 3,2 | RNABind | 215 | 1,1 | - |
| 120 | 0,0006 | - | 1236 | 3,0 | - | 90 | 0,9 | - |
| 326 | 0,0006 | - | 1229 | 3,0 | - | 88 | 0,8 | - |
| 1019 | 0,0006 | - | 1231 | 3,0 | - | 91 | 0,8 | - |
| 258 | 0,0009 | - | 1238 | 2,9 | - | 126 | 0,7 | - |
| 259 | 0,0012 | - | 1230 | 2,9 | - | 212 | 0,7 | - |
| 579 | 0,0012 | - | 1228 | 2,8 | - | 33 | 0,7 | - |
| 297 | 0,0014 | - | 1241 | 2,7 | - | 131 | 0,6 | - |
| 1168 | 0,0015 | - | 1240 | 2,5 | - | 213 | 0,6 | - |
| 266 | 0,0022 | - | 1239 | 2,4 | - | 125 | 0,6 | - |
| 274 | 0,0022 | - | 1227 | 2,4 | - | 127 | 0,5 | - |
| 1167 | 0,0028 | - | 1226 | 2,3 | - | 92 | 0,5 | - |
| 219 | 0,0070 | - | 1244 | 2,2 | - | 132 | 0,5 | - |
| 212 | 0,0076 | - | 1242 | 2,1 | RNABind | 128 | 0,5 | - |
| 228 | 0,0097 | - | 1225 | 2,0 | - | 34 | 0,5 | - |
| 1182 | 0,0165 | - | 1245 | 2,0 | - | 66 | 0,4 | - |

| **Table S13**: Top 20 residues with increased and top 20 residues decreased communication hub importance (Betweenness Centrality) and structural flexibility (RMSF) in the ValRS **H566Y** variant compared to the wild-type. | | | | | | | | |
| --- | --- | --- | --- | --- | --- | --- | --- | --- |
| **Betweenness Centrality** | | | **RMSF (Å)**  (Catalytic) | | | **RMSF (Å)**  (GST-like domain) | | |
| **Position** | **ΔBetweenness**  (WT-MUT) | **Importance** | **Position** | **ΔRMSF**  (WT-MUT) | **Importance** | **Position** | **ΔRMSF**  (WT-MUT) | **Importance** |
| 225 | -0,0099 | - | 895 | -1,3 | - | 49 | -0,9 | - |
| 487 | -0,0056 | - | 897 | -1,3 | - | 37 | -0,9 | - |
| 263 | -0,0038 | - | 896 | -1,3 | - | 36 | -0,8 | - |
| 231 | -0,0024 | - | 893 | -1,2 | - | 48 | -0,7 | - |
| 230 | -0,0022 | - | 747 | -1,2 | - | 47 | -0,7 | - |
| 486 | -0,0020 | - | 894 | -1,2 | - | 46 | -0,6 | - |
| 404 | -0,0012 | - | 898 | -1,1 | - | 50 | -0,5 | - |
| 258 | -0,0009 | - | 746 | -1,0 | - | 45 | -0,5 | - |
| 1128 | -0,0006 | - | 748 | -1,0 | - | 38 | -0,5 | - |
| 741 | -0,0004 | - | 892 | -1,0 | - | 31 | -0,5 | - |
| 1233 | -0,0003 | - | 900 | -1,0 | - | 35 | -0,4 | - |
| 743 | -0,0003 | - | 899 | -0,9 | - | 29 | -0,4 | - |
| 254 | -0,0003 | - | 901 | -0,9 | - | 51 | -0,3 | - |
| 229 | -0,0003 | - | 891 | -0,9 | - | 32 | -0,3 | - |
| 560 | -0,0002 | - | 890 | -0,8 | - | 30 | -0,2 | - |
| 744 | -0,0002 | - | 745 | -0,8 | - | 44 | -0,2 | - |
| 740 | -0,0002 | - | 889 | -0,7 | - | 39 | -0,1 | - |
| 1036 | -0,0002 | - | 333 | -0,7 | - | 27 | -0,1 | - |
| 1231 | -0,0002 | - | 902 | -0,7 | - | 28 | -0,1 | - |
| 495 | -0,0002 | - | 904 | -0,7 | - | 43 | -0,1 | - |
| 30 | 0,0003 | - | 1234 | 2,8 | - | 218 | 2,3 | - |
| 330 | 0,0003 | - | 1233 | 2,7 | - | 88 | 2,1 | - |
| 1083 | 0,0003 | - | 1232 | 2,6 | RNABind | 217 | 2,0 | - |
| 325 | 0,0004 | - | 1235 | 2,4 | RNABind | 125 | 1,9 | - |
| 147 | 0,0005 | - | 1237 | 2,4 | - | 87 | 1,9 | - |
| 48 | 0,0006 | - | 1229 | 2,2 | - | 89 | 1,8 | - |
| 326 | 0,0006 | - | 1231 | 2,2 | - | 123 | 1,8 | - |
| 1019 | 0,0006 | - | 1238 | 2,2 | - | 124 | 1,8 | - |
| 120 | 0,0006 | - | 1236 | 2,2 | - | 126 | 1,7 | - |
| 259 | 0,0007 | - | 1230 | 2,1 | - | 215 | 1,4 | - |
| 579 | 0,0013 | - | 1228 | 2,1 | - | 216 | 1,3 | - |
| 297 | 0,0014 | - | 1241 | 1,9 | - | 86 | 1,2 | - |
| 266 | 0,0024 | - | 561 | 1,9 | - | 122 | 1,2 | - |
| 1167 | 0,0028 | - | 1240 | 1,8 | - | 127 | 1,1 | - |
| 274 | 0,0032 | - | 1239 | 1,8 | - | 90 | 0,9 | - |
| 1168 | 0,0038 | - | 524 | 1,8 | - | 131 | 0,9 | - |
| 219 | 0,0070 | - | 562 | 1,8 | - | 120 | 0,9 | - |
| 212 | 0,0076 | - | 526 | 1,8 | - | 121 | 0,9 | - |
| 228 | 0,0080 | - | 488 | 1,8 | - | 119 | 0,8 | - |
| 1182 | 0,0165 | - | 1227 | 1,7 | - | 85 | 0,8 | - |

| **Table S14**: Root mean square deviation (RMSD) analysis across ValRS functional domains for wild-type and mutant systems. RMSD values are reported in angstroms (Å). | | | | | | | | | |
| --- | --- | --- | --- | --- | --- | --- | --- | --- | --- |
| Domains | **WT** | **L16R** | **P51S** | **M296V** | **R404Q** | **H566Y** | **R665W** | **S866C** | **D920N** |
| **GST** | 2.9±0.6 | 2.8±0.4 | 3.8±1.0 | 3.3±0.5 | 3.3±1.0 | 2.8±0.5 | 3.2±0.4 | 3.1±0.7 | 3.4±0.7 |
| **CP1** | 1.2±0.2 | 1.2±0.2 | 1.6±0.3 | 1.6±0.3 | 1.3±0.2 | 1.3±0.4 | 1.5±0.3 | 1.2±0.2 | 1.2±0.2 |
| **RF** | 2.4±0.6 | 2.7±0.9 | 2.6±0.5 | 2.2±0.6 | 2.8±0.5 | 2.3±0.6 | 1.9±0.5 | 2.4±0.7 | 2.6±0.7 |
| **Anticodon** | 4.2±1.7 | 4.3±0.9 | 3.1±0.7 | 4.1±1.0 | 3.7±0.9 | 3.8±1.0 | 4.8±1.1 | 4.7±1.1 | 4.2±1.2 |
| **Catalytic**  (CP1+RF+Anticodon) | 2.9±1.0 | 3.7±1.5 | 3.2±0.7 | 2.8±0.5 | 3.2±0.7 | 3.0±0.7 | 2.3±0.5 | 2.8±0.5 | 2.9±0.9 |

| **Table S15:** Pearson correlation coefficients of RMSF profiles between independent replicate MD simulations | | | | | |
| --- | --- | --- | --- | --- | --- |
| **System** | **Domain** | **Rep1-2** | **Rep1-3** | **Rep2-3** | **Mean** |
| WT | Catalytic | 0.95 | 0.91 | 0.90 | 0.92 ± 0.02 |
|  | GST-like | 0.90 | 0.91 | 0.94 | 0.92 ± 0.02 |
| R404Q | Catalytic | 0.91 | 0.96 | 0.92 | 0.93 ± 0.02 |
|  | GST-like | 0.88 | 0.94 | 0.94 | 0.92 ± 0.02 |
| H566Y | Catalytic | 0.95 | 0.92 | 0.96 | 0.94 ± 0.02 |
|  | GST-like | 0.94 | 0.93 | 0.96 | 0.94 ± 0.02 |
| D920N | Catalytic | 0.89 | 0.90 | 0.83 | 0.87 ± 0.03 |
|  | GST-like | 0.91 | 0.86 | 0.94 | 0.90 ± 0.03 |

| **Table S16:** Root mean square deviation (RMSD) analysis across ValRS systems with replica MD runs (3 x 200ns). RMSD values are reported in angstroms (Å). | | | | | |
| --- | --- | --- | --- | --- | --- |
| **System** | **Domain** | **Rep1** | **Rep2** | **Rep3** |  |
| WT | Catalytic | 2.8±0.5 | 3.3±1.4 | 2.6±0.4 |  |
|  | GST-like | 3.0±0.7 | 2.8±0.4 | 3.0±0.5 |  |
| R404Q | Catalytic | 3.1±0.6 | 3.4±0.9 | 3.1±0.5 |  |
|  | GST-like | 2.5±0.5 | 3.8±0.9 | 3.7±0.9 |  |
| H566Y | Catalytic | 2.9±0.6 | 2.6±0.5 | 3.4±0.7 |  |
|  | GST-like | 2.7±0.5 | 2.6±0.6 | 2.9±0.5 |  |
| D920N | Catalytic | 2.5±0.5 | 2.8±0.7 | 3.4±1.2 |  |
|  | GST-like | 3.1±0.7 | 3.4±0.6 | 3.7±0.8 |  |

**Figure S1**: **Predicted ValRS (*H. sapiens*) structural models (Alphafold2 - purple, AlphaFold3 - green) aligned with the experimentally resolved ValRS (*T. thermophilus*) (PDB code: 1GAX - red).** (A) Full length structural alignment, (B) Residues of the aminoacylation center alignment, (C) Residues of the editing center alignment (D, E) Residues of the tRNA binding domain center alignment. Residue labels in B-E refer to the human protein (see also Table S3).

**Figure S2**: **ValRS structural models.** (A) Experimentally resolved *T. thermophilus* protein complex with its tRNA (PDB code: 1GAX), (B) AlphaFold3 prediction of *H. sapiens* protein sequence (UniProt ID: P26640) with *H. sapiens* tRNA (tRNA-Val-TAC-1-1), (C) While AlphaFold3 prediction is sufficiently accurate for the protein structure (pLDDT: 89.33 ± 10.81), the positioning of the tRNA is less confident (pLDDT: 65.71 ± 4.03). After alignment with the bacterial structure, important contacts like the anticodon region and the amino acid attachment site deviate from the resolved homologue structure.

**Figure S3**: **Molecular dynamics analysis of the ValRS P51S variant.** (A) RMSD trajectories across the four protein domains. (B) Residue-level dynamic cross-correlation map (upper triangle: wild-type; lower triangle: P51S mutant). (C) Dynamic binding analysis yielded an average ΔG of −7.7 ± 0.5 kcal/mol.

**Figure S4**: **Molecular dynamics analysis of the ValRS R404Q variant.** (A) RMSD trajectories across the four protein domains over the three independent replica runs. (B) Residue-level dynamic cross-correlation map (upper triangle: wild-type; lower triangle: R404Q mutant). (C) Dynamic binding analysis yielded an average ΔG of −7.8 ± 0.5 kcal/mol over the three replica runs.

**Figure S5**: **Molecular dynamics analysis of the ValRS R665W variant.** (A) RMSD trajectories across the four protein domains. (B) Residue-level dynamic cross-correlation map (upper triangle: wild-type; lower triangle: R665W mutant). (C) Dynamic binding analysis yielded an average ΔG of −7.9 ± 0.5 kcal/mol.

**Figure S6**: **Molecular dynamics analysis of the ValRS S866C variant.** (A) RMSD trajectories across the four protein domains. (B) Residue-level dynamic cross-correlation map (upper triangle: wild-type; lower triangle: S866C mutant). (C) Dynamic binding analysis yielded an average ΔG of −7.9 ± 0.4 kcal/mol.

**Figure S7**: **Molecular dynamics analysis of the ValRS D920N variant.** (A) RMSD trajectories across the four protein domains over the three independent replica runs. (B) Residue-level dynamic cross-correlation map (upper triangle: wild-type; lower triangle: D920N mutant). (C) Dynamic binding analysis yielded an average ΔG of −7.9 ± 0.5 kcal/mol over the three replica runs.

**Figure S8**: **Molecular dynamics analysis of the ValRS H566Y variant.** (A) RMSD trajectories across the four protein domains over the three independent replica runs. (B) Residue-level dynamic cross-correlation map (upper triangle: wild-type; lower triangle: H566Y mutant). (C) Dynamic binding analysis yielded an average ΔG of −7.9 ± 0.6 kcal/mol over the three replica runs.

**Figure S9**: Root mean square fluctuation (RMSF) analysis revealing residue-level flexibility changes upon mutation.

**1. SUPPLEMENTARY METHODS**

**1.1 Exome sequencing**

Genomic DNA was extracted from peripheral blood samples, according to standard procedures of phenol-chloroform extraction.  Exome sequencing on DNA of subjects was performed as described elsewhere in Macrogen, Korea. Briefly, target enrichment was performed with 2 μg genomic DNA using the SureSelectXT Human All Exon Kit version 6 (Agilent) to generate barcoded exome sequencing libraries. Libraries were sequenced on the HiSeqX platform (Illumina) with 50x coverage. Quality assessment of the sequence reads was performed by generating QC statistics with FastQC. The bioinformatics filtering strategy included screening for only exonic and donor/acceptor splicing variants [6].

**1.2 Evolutionary-Guided Sequence analysis**

1.2.1 Multiple Sequence Alignment (MSA) and Conservation Analysis

To identify evolutionarily conserved residues within the full-length protein sequence, MSA was performed using the ConSurf web server [7–11]. Homologous sequences were retrieved from the UniProt90 database using a single iteration of the HMMER search algorithm [12] with an E-value cutoff of 0.0001. To ensure meaningful evolutionary comparison, homologues were filtered to retain sequences with 35–95% identity to the query. From the resulting pool, 500 sequences were randomly selected for downstream analysis. Sequence alignment was conducted using the MAFFT-L-INS-i algorithm [13], optimized for accuracy in aligning sequences with conserved local motifs. Evolutionary conservation scores were then computed using the Bayesian method as implemented in ConSurf. All visualization and analysis steps were carried out through the ConSurf web interface.

1.2.2 Homology-Based Domain Annotation and Structural Inference

To investigate the functional domains and structural elements of the human valyl-tRNA synthetase (UniProt ID: P26640), we initially consulted the InterPro database [14], which provided domain annotations based on sequence analysis. However, due to the lack of an experimentally resolved structure for the human protein (as of June 2025), we turned to homologous bacterial structures as a reference. Specifically, we curated high-resolution crystallographic structures of *Thermus thermophilus* valyl-tRNA synthetase from the Protein Data Bank (PDB IDs: 1GAX [15], 1IVS [16], 1IYW [16], 1WK9 [17], 1WKA [17]), which offer detailed insights into functionally important regions. From these structures, we identified key amino acids and motifs involved in enzymatic function and structural integrity. To transfer this knowledge to the human protein, we aligned the bacterial sequence to the human sequence using BLASTp [18], with the human sequence as the query. This allowed us to map the structurally and functionally relevant elements from the bacterial protein onto the human sequence, enabling a residue-level interpretation of potentially important features in the human protein despite the absence of experimentally resolved structural data.

**1.3 Acquisition of Structure, *In Silico* Mutagenesis and Simulation in Physiological Conditions**

As no experimental 3D structure was available, the predicted structure of the human valyl-tRNA synthetase was retrieved from the AlphaFold2 (AF2) database [19–21], which models the full-length protein in its apo state. Although AlphaFold3 (AF3) [22] also offers bound-state predictions, including a complex with the cognate tRNA (tRNA-Val-TAC-1-1) and adenosine monophosphate (AMP) substrate, this model was only used for identifying potentially important interaction regions and was not included in the simulations. The AF2-predicted structure was subjected to all-atom molecular dynamics (MD) simulations in its unbound form to allow for structural relaxation and naturalistic sampling. Three independent replicas of 200 ns were performed. Each system was solvated in a rectangular box using TIP3P water [23] and 0.15 M NaCl, constructed via CHARMM-GUI [24–26] with a 12 Å buffer from the protein surface, resulting in simulation systems of approximately 770,000 atoms. The trajectories from the three replicas were merged and clustered using GROMOS [27] with an RMSD threshold of 1.0 Å to identify dominant conformations. A representative centroid structure was selected and further simulated for an additional 200 ns. A final round of GROMOS clustering (RMSD threshold = 1.0 Å) was then conducted to obtain the definitive representative structure used for downstream mutation modeling.

To investigate the effects of disease-associated and novel variants, selected point mutations were introduced into the final wild-type structure using Schrödinger Maestro molecular modeling package. Each mutant was solvated, minimized, equilibrated, and simulated for another 200 ns, following the same protocol as the wild-type system, allowing for direct comparison of structural stability, flexibility, and local rearrangements.

All simulations were performed using GROMACS 2023.3 [28]. Systems were first subjected to energy minimization via the steepest descent algorithm for 10,000 steps. Equilibration was conducted under combined NVT/NPT conditions using a V-rescale thermostat (set at 310 K) and a C-rescale barostat (set at 1 atm). Nonbonded interactions were treated using a 12 Å cutoff for van der Waals and short-range electrostatics, while long-range electrostatics were computed using the Particle Mesh Ewald (PME) method. Production runs were executed using the CHARMM36m force field [29]. Due to the large system size and computational constraints a single 200 ns MD simulations were performed for each mutant system. To validate the reproducibility of our findings, four representatives out of the nine systems (wild-type, R404Q, H566Y, and D920N) were subjected to two additional independent replica simulations of 200 ns each (three runs per system, 600 ns total per system).

**1.4 Trajectory analysis**

1.4.1 Root Mean Square Deviation (RMSD) and Root Mean Square Fluctuation (RMSF)

To assess the structural stability of each system, RMSD analysis was performed using VMD, focusing on the backbone atoms of the protein across the entire MD trajectory. The last frame of equilibration was used as the reference structure. The RMSD at time t was computed using the standard equation 1:

$$RMSD= \sqrt{\frac{\sum_{i=1}^{N} \left( x_{i}-\hat{x}_{i} \right)^{2}}{N}} (1)$$

Where $x_{i}$ is the data point at i^th^ position in each examined frame, and $\hat{x}_{i}$ is the data point at i^th^ position in the reference frame. RMSD was calculated per individual domains separately.

In addition to RMSD, RMSF analysis was performed using VMD to quantify per-residue atomic fluctuations. RMSF values were calculated relative to the same reference structure, offering insights into local flexibility across the protein and how each mutation reshaped the residue-specific dynamic profile, either preserving or altering wild-type-like behavior.

1.4.2 Principal Component Analysis and Clustering

To characterize large-scale collective motions within the enzymatic core of the protein, PCA was applied to the MD trajectories. PCA was performed on the Cartesian coordinates of Cα atoms, allowing for dimensionality reduction of the trajectory and identification of dominant conformational motions. The covariance matrix of atomic positional fluctuations was constructed from the aligned trajectory, and eigenvalues and eigenvectors were computed using the PCA module from scikit-learn [30]. The top principal components (PCs) were visualized using Matplotlib to reveal dominant structural transitions. This analysis enabled the identification of conformational clusters sampled during the simulation of the AF2-predicted apo structure.

1.4.3 Network-Based Analysis of Residue Communication

Network analysis of intra-protein communication was performed using betweenness centrality (BC), a graph-theoretical measure that identifies residues acting as communication hubs within the protein structure. Residue interaction networks (RINs) were constructed from MD trajectories using MD-TASK, where each residue was represented as a node, and edges were defined between pairs of residues with Cβ-Cβ (or Cα for glycine) distances within 7.0 Å over the course of the trajectory. Betweenness centrality was calculated for each residue as the fraction of shortest paths in the network that traverse through it. Formally, for a residue 𝑣, BC is defined as in equation 2:

$${BC}_{v}= \sum_{s, t \epsilon V} \frac{\sigma\left( s,t \right| v)}{\sigma(s,t)} (2)$$

where σ is the total number of shortest paths between residues s and t. Residues with high BC values are considered critical for maintaining efficient communication across distant regions of the protein and often coincide with sites involved in allosteric regulation, interdomain coupling, or catalytic function. Final BC scores underwent normalization to range between -1 and +1.

We utilized MD-TASK [31] to build the protein networks and calculate per residue BC values for both the wild-type and mutant simulations. BC was calculated as the fraction of shortest communication paths traversing each node, identifying key residues involved in intra-protein signaling. In other words it shows how important a residue is for communication between other residues in the protein, acting like a bridge in the network of interactions. Mutant-induced shifts in BC values were compared to the wild type to detect altered communication hubs or bottlenecks. This allowed the identification of long-range effects of mutations on interdomain communication, particularly in regions critical for protein function.

1.4.4 Dynamic Cross-Correlation Analysis of Residue Motions

To evaluate the impact of mutations on residue-residue coordination, we computed DCCMs using MD-TASK [31]. DCCMs quantify the extent to which atomic displacements of residue pairs are correlated throughout the MD trajectory. Correlation coefficients were calculated using Cα atomic positions, resulting in values ranging from -1 to +1. A value of +1 indicates perfect concerted motion, -1 indicates perfect anti-correlated motion, and values near 0 reflect uncorrelated behavior. The DCCM provides a symmetric matrix in which each element represents the correlation of atomic displacements between a pair of residues over time. These matrices were visualized as heatmaps using Matplotlib and Seaborn in Python [32,33]. Correlation patterns were analyzed to detect clusters of coordinated motion (strong positive correlations) and regions of decoupling (reduced or negative correlations). Comparative analysis between mutant variants and the wild-type structure highlighted alterations in dynamic coupling, particularly within and between key functional domains such as the GST-like region, the catalytic core, and RNA-binding motifs.

1.4.5 Dynamic Binding Analysis throughout Trajectory

To evaluate how mutations affect ligand binding throughout the simulation, we developed a custom dynamic docking pipeline, designed to quantify binding affinity changes over time directly from MD trajectories. At its core, the method integrates smina [34] as the docking engine, embedded within a Python-based workflow that is developed by our research group that automates all aspects of frame extraction, receptor preparation, ligand docking, and scoring.

In our implementation, snapshots were extracted from the catalytic domain of each trajectory at regular intervals of 5 frames. Frames were then aligned to a reference conformation, and the receptor was automatically prepared with the integrated MGLTools, and local grid setup around the active site was generated (box size of 15Å). The prepared ATP-valyl ligand was docked onto each frame using smina, and both the binding affinity score and ligand RMSD (with respect to the original reference pose) were computed for each time point. The resulting time-resolved profiles were visualized to assess how ligand binding energy and pose stability evolve throughout the trajectory. This approach enabled us to systematically track dynamic changes in ligand interaction behavior, particularly to assess whether specific mutations disrupted the tRNA charging ability of the protein.

**2. SUPPLEMENTARY RESULTS**

**2.1 Structural Interpretation of ValRS Mutations**

To dynamically investigate the structural impact of patient-derived mutations in ValRS, we employed our MD-refined apo model to map the spatial proximity of each variant to conserved residues involved in three key functional regions: the valine-binding pocket (aminoacylation), the threonine-editing site (proofreading), and the tRNA-binding interface. This spatial analysis was integrated with AlphaFold-based pathogenicity scores to assess the potential functional consequences of each mutation.

Within the Valine-binding pocket, residue R665 is situated near residue 345 (6.4 Å), while residue S866 lies within the conserved KMSKS motif and is 6.6 Å from residue 863, both contributing to Valine recognition and ligand entry/exit dynamics. The KMSKS motif is a hallmark of class I aminoacyl-tRNA synthetases and plays a pivotal role in substrate binding. AlphaFold predicts both R665W and S866C mutations to be likely pathogenic, with scores of 0.9873 and 0.9072, respectively. Accordingly, in the CP1 editing domain, H566 is located 8.7 Å from residue 591, a core component of the threonyl-tRNA^Val hydrolysis site. The H566Y substitution likely introduces steric hindrance or shifts in local polarity within this spatially constrained environment. Consistent with this, AlphaFold2 classifies the variant as likely pathogenic (score: 0.7252).

The tRNA-binding surface harbors the highest density of spatially proximal variants. M296 resides in this interface, with close contacts to residues 958 (3.0 Å), 955 (6.6 Å), 959 (6.8 Å), and 951 (8.7 Å). Despite the conservative nature of the M296V substitution, it is predicted to be likely benign (score: 0.228), suggesting limited structural disruption, potentially affecting peripheral tRNA accommodation rather than catalytic function. In contrast, R404, located in the Rossmann fold domain, does not directly interact with any catalytic residues, but its mutation to glutamine (R404Q) is predicted to be likely pathogenic (score: 0.906), possibly due to altered surface charge distribution or disruption of interdomain communication. D920, positioned within 6.3-6.8 Å of residues 951 and 947, both involved in RNA anchoring, likely contributes to a stabilizing polar interaction or salt bridge. Its mutation to asparagine (D920N), though chemically similar, is predicted to be pathogenic (score: 0.9758), likely due to impaired electrostatic stabilization.

Finally, mutations in the GST-like domain, L16R and P51S, are spatially distant from the active, editing, and tRNA-binding sites. The L16R substitution introduces a charged residue into a hydrophobic environment and is predicted to be benign (score: 0.3374). Similarly, P51S likely results in a modest reduction in local rigidity without major energetic penalties (score: 0.0808). While these changes are unlikely to disrupt catalysis or substrate recognition directly, they may subtly influence domain folding or interactions within the multisynthetase complex. It is important to note that these assessments are based on static spatial proximity and structure-based pathogenicity predictions. They do not capture long-range conformational coupling, dynamic rearrangements, or allosteric regulation, which may become evident only under physiologically relevant motions. These dynamic aspects are further explored in the subsequent section through MD simulations.

**2.2 Functional Interpretation of ValRS Mutations with MD simulations**

2.2.1 Mutations on the GST-like domain

The GST-like domain, although not directly involved in the catalytic activity of the enzyme, plays a crucial structural and scaffolding role by mediating protein–protein interactions and stabilizing the spatial organization of the adjacent domains [35]. Mutations within this region, are not typically expected to cause pronounced local destabilization due to the domain’s compact and well-folded nature but can exert long-range influence on the global conformational behavior and functional coordination of the protein.

**P51S** substitution of Proline to Serine at position 51 resides within the GST-like domain. During the MD simulations, this mutation resulted in a slight increase in structural deviation of the GST-like domain, with the mutant displaying an RMSD of 3.8 ± 1.0 Å compared to 2.9 ± 0.6 Å in the wild type, which suggests that the mutation introduces local flexibility or partial destabilization in this structurally important domain. Although the catalytic core also exhibited slightly elevated fluctuations (RMSD: 3.2 ± 0.7 Å in the mutant vs. 2.9 ± 1.0 Å in the wild type), the effect was less pronounced than in the GST domain. Among specific subdomains, the anticodon-binding region exhibited minor flexibility increase in the mutant (RMSD: 3.1 ± 0.7 Å) from the wild type (4.2 ± 1.7 Å), while showcased similar fluctuations in the Rossmann fold domain (2.6 ± 0.5 Å in mutant vs. 2.4 ± 0.6 Å in WT) and in the CP1 editing domain (1.6 ± 0.3 Å in mutant vs. 1.2 ± 0.2 Å in WT) (Table S14). Correlation analysis revealed an increase in internal correlation within both the GST-like and catalytic domains in the mutant, potentially reflecting a tighter or altered coupling between neighboring residues induced by the mutation (Figure S3B).

Revealed by the MD simulation, we can suggest deviation in the communication network among the residues from the wild type, promoted by the mutated phenotype (Table S7). Among which we focused on residue Aps584, which exhibited a small increase in betweenness centrality (ΔBC = -0.0014) and residue Met583 showing a similar but milder trend (ΔBC = -0.0004). These residues are positioned within the threonine recognition region of the CP1 domain and changes in their communication role and / or hub importance which suggest mild perturbations in protein function. Moreover, measures of residue fluctuations with RMSF also revealed decrease of the region around Pro1232 (+2.9 Å) and Val1235 (+2.6 Å) in the mutant from the wild type, two residues important for the tRNA binding (Table S7). On the contrary, no residues important for ATP-valyl ligand binding seemed to be impacted by the alteration, as its shown also by the stable docking energies observed between the wild type (-7.9 ± 0.5 kcal/mol) and mutant (-7.7 ± 0.5 kcal/mol), indicating that ATP-valyl ligand recognition and binding strength were preserved (Figure S3C).

2.2.2 Mutations on the main catalytic domain

The main catalytic domain constitutes the enzymatic core of the protein, housing the aminoacylation active site and playing a central role in tRNA recognition and amino acid charging. Due to its direct involvement in catalysis, mutations within this region are more likely to induce functional and structural consequences [15,16,36,37].

**R404Q.** Position 404 lies within the catalytic domain, positioned near the interface critical for ATP-valyl and tRNA interaction. Over the three replica simulation trajectories, the GST-like domain exhibited similar flexibility in the mutant (RMSD: 3.3 ± 1.0 Å) compared to the wild type (2.9 ± 0.6 Å), as also did the catalytic domain (3.2 ± 0.7 Å in mutant vs. 2.9 ± 1.0 Å in WT). Specifically, the anticodon-binding domain displayed 3.7 ± 0.9 Å in mutant relative to 4.2 ± 1.7 Å in the wild type and the CP1 and Rossmann fold domains remained largely stable with minimal differences (1.3 ± 0.2 Å, 2.8 ± 0.5 Å in mutant vs. 1.2 + 0.2 Å, 2.4 ± 0.6 Å in WT, accordingly) (Table S14). Cross-correlation analysis revealed reduction within the catalytic domain, suggesting some residue-specific flexibility (Figure S4B).

At the local interaction level, the mutation involves a substitution from a positively charged arginine to a neutral polar glutamine, which is expected to induce side-chain repacking in its immediate neighborhood. Intrestingly though, there was no measurable deviation in any of the important residue hubs from the wild type (in all three independent replica runs). However, ΔRMSF from mutant to wild type suggested decreased fluctuation in residues Pro1232 (+3.6 Å), Val1235 (+3.2 Å) and Lys1242 (+2.0 Å) important in tRNA binding (Table S9). Once again, the dynamic binding analysis suggested that the ATP-valyl binding energy remained almost identical between mutant and wild type (-7.8 ± 0.5 kcal/mol in mutant vs. -7.9 ± 0.5 kcal/mol in WT), indicating preserved ligand affinity at the active site (Figure S4C).

Taken together, the R404Q mutation leads to moderate dynamic changes within the catalytic core and nearby RNA-binding regions. The pathogenic R404W variant, a previously described mutation at the same site, has been experimentally linked to partial loss of aminoacylation activity, though the underlying mechanism remains uncharacterized [4,38]. Our simulations suggest that perturbation of tRNA positioning, rather than direct disruption of valine binding, may underlie the functional deficit at this site, however such a hypothesis needs further experimental validation through tRNA binding assays and kinetic studies.

**R665W.** Residue at position 665 is positioned within the catalytic domain. Over the simulation, the GST-like and the catalytic domains in the mutant appeared similarly flexible (RMSD: 3.2 ± 0.4 Å, 2.3 ± 0.5 Å, accordingly) with the wild type (2.9 ± 0.6 Å, 2.9 ± 1.0 Å, accordingly) . More analytically for the catalytic domains: anticodon-binding domain (4.8 ± 1.1 Å in mutant vs. 4.2 ± 1.7 Å in WT), the CP1 editing domain (1.5 ± 0.3 Å in mutant vs. 1.2 ± 0.2 Å in WT) and the Rossmann fold domain (1.9 ± 0.5 Å in mutant vs. 2.4 ± 0.6 Å WT) mobility was similar in both phenotypes (Table S14).

Regarding the amino acid communication network, one residue Met583 (ΔBC = -0.0002) important for the threonine selectivity mechanism appeared more important for the protein network. At the same time, the close-by residue Asp584 (ΔRMSF: -0.5 Å), also important from the substrate selectivity, found to fluctuate more than in the mutant. In addition, residues Pro1232 (+4.7 Å), Val1235 (+4.2 Å) and Lys1242 (+2.2 Å) important in tRNA binding appeared to fluctuate less in the mutant than in the wild type (Table S10). The ATP-valyl binding affinity remained once again unchanged (-7.9 ± 0.5 kcal/mol for both wild type and mutant), indicating that ligand recognition by the catalytic core is energetically preserved despite the mutation-induce structural deviations (Figure S5C).

**S866C.** This mutation is positioned within the conserved KMSKS loop of the catalytic domain, a strongly conserved motif as seen in the multiple sequence alignments we performed (Table S4). During the MD simulation both the GST-like (RMSD: 3.1 ± 0.7 Å) and catalytic (2.8 ± 0.5 Å) domains retained similar flexibility with the wild type (2.9 ± 0.6 Å, 2.9 ± 1.0 Å accordingly), Specifically, the deviation observed was almost identical: anticodon-binding domain (4.7 ± 1.1 Å in mutant vs. 4.2 ± 1.7 Å in WT), CP1 editing domain (1.2 ± 0.2 Å in both) and Rossmann fold domain remained stable (2.4 ± 0.6 Å in both) (Table S14). Cross-correlation analysis also revealed no major alterations in overall coupling patterns, with GST and catalytic domains displaying similar internal correlations in both wild type and mutant systems (Figure S6B).

On the residue level, the mutation seemed to have preserved the global RMSF profile similar to the wild type one with no any of the important residues to be affected (not more than ±1.0 Å). However, our data suggests that the mutation has increased the importance of one communication hub, this of residue Pro1232 (ΔBC= -0.002) important for tRNA binding (Table S11). Still, the ATP-valyl substrate binding energy remained unchanged between wild type and mutant (-7.9 ± 0.5 kcal/mol in both), suggesting that ligand recognition per se is not directly affected (Figure S6C).

**D920N.** The D920N mutation is located within the catalytic domain and in proximity to residues essential for coordinating the tRNA substrate (Figure S1D-E). Over the MD simulations, the GST-like domain displayed comparable dynamics (RMSD: 3.4 ± 0.7 Å in mutant vs. 2.9 ± 0.6 Å in WT), while the catalytic domain exhibited almost identical dynamical fluctuations in both phenotypes (2.9 ± 0.9 Å in mutant vs. 2.9 ± 1.0 Å in WT). All subdomains of the catalytic part also remained individually unaffected (Table S14). Cross-correlation analysis revealed similar dynamic coupling across both GST-like domain and a slight decrease in internal correlation for the mutant’s catalytic domain (Figure S7B).

Regarding protein communication network deviations between the mutation and wild type phenotypes Pro1232 (ΔBC= -0.0035) important for tRNA binding was once again revealed to have increased hub importance, while also Asp584 (ΔBC= -0.0004) residue contributing to threonine selectivity was reported to have slightly become more important hub in the mutation dynamics. Moreover, residues Pro1232 (+3.4 Å), Val1235 (+3.2 Å) and Lys1242 (+2.1 Å) contributing to tRNA binding appeared to fluctuate less in the mutant than in the wild type (Table S12). The ATP-valyl binding affinity remained stable between wild type and mutant (ΔG = -7.9 ± 0.5 kcal/mol in both cases), proposing that neither this mutation does not energetically perturb ATP substrate recognition (Figure S7C).

2.2.3 Mutations on the editing domain

The edinting domain (CP1) ensures fidelity during aminoacylation by removing mischarged amino acids. In valRS, specifically, it ensures the tRNA will not be charged with threonine instead of valine. Beyond this selectivity function, the CP1 domain acts allosterically to modulate the specificity and activity of the catalytic core [39]. Thus, mutations within this domain may alter editing efficiency or perturb long-range communication networks, even when catalytic and ligand-binding capacities remain intact.

**H566Y** This mutation resides within the CP1 editing domain. During the MD simulations, both the mutated protein’s GST-like (RMSD: 2.8 ± 0.5 Å), as well as the catalytic part (3.0 ± 0.7 Å), displayed similar fluctuation compared to the wild type protein’s domains (2.9 ± 0.6 Å, 2.9 ± 11.0 Å, accordingly). In the catalytic part, the anticodon-binding domain showed the maximum deviation (though not significant) across the two phenotypes (3.8 ± 1.0 Å in mutant vs. 4.2 ± 1.0 Å in WT), while the CP1 and Rossmann fold domains presented almost identical fluctuation rate (about 1.2 - 2.4 Å) (Table S14). Minor shifts were observed in cross-correlation analysis: while the GST domain maintained its dynamic coherence, the catalytic domain showed reduced internal correlation in the mutant (Figure S8B).

In the residue level, there was no measurable deviation in any of the important residue hubs from the wild type but RMSF comparison of mutant and wild type suggested reduced fluctuation in residues Pro1232 (+2.6 Å) and Val1235 (+2.4 Å) important in tRNA binding (Table S13). Despite that, ligand binding to the ATP-valyl site remained still unaffected in the dynamic binding analysis, with energies with identical values between wild type and mutant (-7.9 ± 0.6 kcal/mol), suggesting once again that core catalytic function is preserved (Figure S8C).

**3. REFERENCES**

1. Okur V, Ganapathi M, Wilson A *et al.* Biallelic variants in VARS in a family with two siblings with intellectual disability and microcephaly: case report and review of the literature. *Molecular Case Studies* 2018;4:a003301.

2. Siekierska A, Stamberger H, Deconinck T *et al.* Biallelic VARS variants cause developmental encephalopathy with microcephaly that is recapitulated in vars knockout zebrafish. *Nature Communications 2019 10:1* 2019;10:708-.

3. Friedman J, Smith DE, Issa MY *et al.* Biallelic mutations in valyl-tRNA synthetase gene VARS are associated with a progressive neurodevelopmental epileptic encephalopathy. *Nature Communications 2019 10:1* 2019;10:707-.

4. Karaca E, Harel T, Pehlivan D *et al.* Genes that Affect Brain Structure and Function Identified by Rare Variant Analyses of Mendelian Neurologic Disease. *Neuron* 2015;88:499–513.

5. Hiz S, Kiliç S, Bademci G *et al.* VARS1 mutations associated with neurodevelopmental disorder are located on a short amino acid stretch of the anticodon-binding domain. *Turk J Biol* 2022;46:458–64.

6. Rosello M, Caro-Llopis A, Orellana C *et al.* Hidden etiology of cerebral palsy: genetic and clinical heterogeneity and efficient diagnosis by next-generation sequencing. *Pediatr Res* 2021;90:284–8.

7. Yariv B, Yariv E, Kessel A *et al.* Using evolutionary data to make sense of macromolecules with a “face-lifted” ConSurf. *Protein Science* 2023;32, DOI: 10.1002/pro.4582.

8. Celniker G, Nimrod G, Ashkenazy H *et al.* ConSurf: Using evolutionary data to raise testable hypotheses about protein function. *Isr J Chem* 2013;53:199–206.

9. Ashkenazy H, Erez E, Martz E *et al.* ConSurf 2010: Calculating evolutionary conservation in sequence and structure of proteins and nucleic acids. *Nucleic Acids Res* 2010;38, DOI: 10.1093/nar/gkq399.

10. Ashkenazy H, Abadi S, Martz E *et al.* ConSurf 2016: an improved methodology to estimate and visualize evolutionary conservation in macromolecules. *Nucleic Acids Res* 2016;44:W344–50.

11. Berezin C, Glaser F, Rosenberg J *et al.* ConSeq: The identification of functionally and structurally important residues in protein sequences. *Bioinformatics* 2004;20:1322–4.

12. Potter SC, Luciani A, Eddy SR *et al.* HMMER web server: 2018 update. *Nucleic Acids Res* 2018;46:W200–4.

13. Katoh K, Misawa K, Kuma KI *et al.* MAFFT: a novel method for rapid multiple sequence alignment based on fast Fourier transform. *Nucleic Acids Res* 2002;30:3059–66.

14. Blum M, Andreeva A, Cavalcanti Florentino L *et al.* InterPro: the protein sequence classification resource in 2025. *Nucleic Acids Res* 2025;53:D444–56.

15. Fukai S, Nureki O, Sekine S ichi *et al.* Structural basis for double-sieve discrimination of L-valine from L-isoleucine and L-threonine by the complex of tRNA(Val) and valyl-tRNA synthetase. *Cell* 2000;103:793–803.

16. Fukai S, Nureki O, Sekine SI *et al.* Mechanism of molecular interactions for tRNA(Val) recognition by valyl-tRNA synthetase. *RNA* 2003;9:100–11.

17. Fukunaga R, Yokoyama S. Structural basis for non-cognate amino acid discrimination by the valyl-tRNA synthetase editing domain. *Journal of Biological Chemistry* 2005;280:29937–45.

18. Camacho C, Coulouris G, Avagyan V *et al.* BLAST+: Architecture and applications. *BMC Bioinformatics* 2009;10:1–9.

19. Jumper J, Evans R, Pritzel A *et al.* Highly accurate protein structure prediction with AlphaFold. *Nature 2021 596:7873* 2021;596:583–9.

20. Varadi M, Bertoni D, Magana P *et al.* AlphaFold Protein Structure Database in 2024: providing structure coverage for over 214 million protein sequences. *Nucleic Acids Res* 2024;52:D368–75.

21. Varadi M, Anyango S, Deshpande M *et al.* AlphaFold Protein Structure Database: massively expanding the structural coverage of protein-sequence space with high-accuracy models. *Nucleic Acids Res* 2022;50:D439–44.

22. Abramson J, Adler J, Dunger J *et al.* Accurate structure prediction of biomolecular interactions with AlphaFold 3. *Nature* 2024;630:493–500.

23. Mark P, Nilsson L. Structure and dynamics of the TIP3P, SPC, and SPC/E water models at 298 K. *Journal of Physical Chemistry A* 2001;105:9954–60.

24. Brooks BR, Brooks CL, Mackerell AD *et al.* CHARMM: The biomolecular simulation program. *J Comput Chem* 2009;30:1545–614.

25. Jo S, Kim T, Iyer VG *et al.* CHARMM-GUI: A web-based graphical user interface for CHARMM. *J Comput Chem* 2008;29:1859–65.

26. Lee J, Cheng X, Swails JM *et al.* CHARMM-GUI Input Generator for NAMD, GROMACS, AMBER, OpenMM, and CHARMM/OpenMM Simulations Using the CHARMM36 Additive Force Field. *J Chem Theory Comput* 2016;12:405–13.

27. Scott WRP, Hünenberger PH, Tironi IG *et al.* The GROMOS Biomolecular Simulation Program Package. *Journal of Physical Chemistry A* 1999;103:3596–607.

28. Abraham MJ, Murtola T, Schulz R *et al.* GROMACS: High performance molecular simulations through multi-level parallelism from laptops to supercomputers. *SoftwareX* 2015;1–2:19–25.

29. Huang J, Mackerell AD. CHARMM36 all-atom additive protein force field: Validation based on comparison to NMR data. *J Comput Chem* 2013;34:2135–45.

30. Pedregosa F, Varoquaux G, Gramfort A *et al.* Scikit-learn: Machine Learning in Python Pedregosa, Varoquaux, Gramfort et al. *Journal of Machine Learning Research* 2011;12:2825–30.

31. Brown DK, Penkler DL, Amamuddy OS *et al.* MD-TASK: a software suite for analyzing molecular dynamics trajectories. *Bioinformatics* 2017;33:2768–71.

32. Hunter JD. Matplotlib: A 2D graphics environment. *Comput Sci Eng* 2007;9:90–5.

33. Waskom M. seaborn: statistical data visualization. *J Open Source Softw* 2021;6:3021.

34. Koes DR, Baumgartner MP, Camacho CJ. Lessons learned in empirical scoring with smina from the CSAR 2011 benchmarking exercise. *J Chem Inf Model* 2013;53:1893–904.

35. Guo M, Schimmel P, Yang XL. Functional expansion of human tRNA synthetases achieved by structural inventions. *FEBS Lett* 2010;584:434.

36. Sugiura I, Nureki O, Ugaji-Yoshikawa Y *et al.* The 2.0 Å crystal structure of Thermus thermophilus methionyl-tRNA synthetase reveals two RNA-binding modules. *Structure* 2000;8:197–208.

37. Nureki O, Vassylyev DG, Tateno M *et al.* Enzyme structure with two catalytic sites for double-sieve selection of substrate. *Science* 1998;280:578–82.

38. Siekierska A, Stamberger H, Deconinck T *et al.* Biallelic VARS variants cause developmental encephalopathy with microcephaly that is recapitulated in vars knockout zebrafish. *Nature Communications 2019 10:1* 2019;10:708-.

39. Li L, Palencia A, Lukk T *et al.* Leucyl-tRNA synthetase editing domain functions as a molecular rheostat to control codon ambiguity in mycoplasma pathogens. *Proc Natl Acad Sci U S A* 2013;110:3817–22.
